# Gasification of coal and biomass as a net carbon-negative power source for environment-friendly electricity generation in China

**DOI:** 10.1073/pnas.1812239116

**Published:** 2019-04-08

**Authors:** Xi Lu, Liang Cao, Haikun Wang, Wei Peng, Jia Xing, Shuxiao Wang, Siyi Cai, Bo Shen, Qing Yang, Chris P. Nielsen, Michael B. McElroy

**Affiliations:** ^a^State Key Joint Laboratory of Environment Simulation and Pollution Control, School of Environment, Tsinghua University, 10084 Beijing, People’s Republic of China;; ^b^State Environmental Protection Key Laboratory of Sources and Control of Air Pollution Complex, Tsinghua University, 10084 Beijing, People’s Republic of China;; ^c^School of Chemical Engineering, The University of Queensland, Brisbane, QLD 4072, Australia;; ^d^State Key Laboratory of Pollution Control and Resource Reuse, School of the Environment, Nanjing University, 210023 Nanjing, People’s Republic of China;; ^e^School of International Affairs, Pennsylvania State University, University Park, PA 16802;; ^f^Department of Civil and Environmental Engineering, Pennsylvania State University, University Park, PA 16802;; ^g^Energy Analysis and Environmental Impacts Division, Lawrence Berkeley National Laboratory, Berkeley, CA 94720;; ^h^Department of New Energy Science and Technology, School of Energy and Power Engineering, Huazhong University of Science and Technology, 430074 Wuhan, People’s Republic of China;; ^i^China-EU Institute for Clean and Renewable Energy, Huazhong University of Science and Technology, 430074 Wuhan, People’s Republic of China;; ^j^John A. Paulson School of Engineering and Applied Sciences, Harvard University, Cambridge, MA 02138;; ^k^Department of Earth and Planetary Sciences, Harvard University, Cambridge, MA 02138

**Keywords:** bioenergy, gasification, CCS, air pollution, carbon-negative energy

## Abstract

Realizing the goal of the Paris Agreement to limit global warming to 2 °C by the end of this century will most likely require deployment of carbon-negative technologies. It is particularly important that China, as the world’s top carbon emitter, avoids being locked into carbon-intensive, coal-fired power-generation technologies and undertakes a smooth transition from high- to negative-carbon electricity production. We focus here on deploying a combination of coal and biomass energy to produce electricity in China using an integrated gasification cycle system combined with carbon capture and storage (CBECCS). Such a system will also reduce air pollutant emissions, thus contributing to China’s near-term goal of improving air quality. We evaluate the bus-bar electricity-generation prices for CBECCS with mixing ratios of crop residues varying from 0 to 100%, as well as associated costs for carbon mitigation and cobenefits for air quality. We find that CBECCS systems employing a crop residue ratio of 35% could produce electricity with net-zero life-cycle emissions of greenhouse gases, with a levelized cost of electricity of no more than 9.2 US cents per kilowatt hour. A carbon price of approximately $52.0 per ton would make CBECCS cost-competitive with pulverized coal power plants. Therefore, our results provide critical insights for designing a CBECCS strategy in China to harness near-term air-quality cobenefits while laying the foundation for achieving negative carbon emissions in the long run.

## Significance

Deploying coal-bioenergy gasification systems with carbon capture and storage (CBECCS) provides a promising opportunity for China to realize its carbon mitigation and air pollution abatement goals simultaneously. We conducted a comprehensive assessment of CBECCS technology for China, with a focus on plant and fuel configurations (e.g., biomass ratios) and economics, as well as CO_2_ and greenhouse gas emissions and cobenefits for air quality. We find significant opportunities for carbon mitigation with air quality cobenefits from deployment of CBECCS systems in regions that are both rich in crop residues and facing urgent needs to curb serious air pollution. The study thus provides critical information for policy makers seeking to exploit the carbon-negative energy opportunities of CBECCS technology.

Deployment of carbon-negative technologies will likely play an important role in achieving long-term carbon mitigation targets. The Paris Agreement on climate change set ambitious targets to hold the increase in the global average temperature to below 2 °C and to pursue efforts to limit the temperature increase to 1.5 °C (1). Many mitigation scenarios have been designed using integrated assessment models to explore possible pathways to achieve the Paris goals. A common feature across all of the climate-stabilization scenarios explored in the 1.5 °C Intergovernmental Panel on Climate Change report (2) is that large-scale application of carbon-negative technologies, especially bioenergy with carbon capture and storage (BECCS), will be necessary in the second half of the century (3). Although the scale of BECCS capacity varies, some deployment of BECCS technology is required in all of these scenarios to achieve deep cuts in greenhouse gas (GHG) emissions (4).

Although the importance of negative emission technologies is widely acknowledged, progress in advancing BECCS deployment has been slow. Given that first-of-a-kind plants will likely be too expensive without substantial government subsidies, cost buy-down and learning by doing has to begin soon for BECCS to be ready for unsubsidized, widespread deployment by midcentury. In addition, due to the difficulty in reversing existing commitments to inexpensive new coal-fired power plants in many developing countries, the need for carbon-negative electricity-generating technologies is even more urgent to offset emissions anticipated from these plants.

Existing studies of BECCS often focus on two technology pathways to convert bioenergy into liquid fuels: (*i*) through biochemical processes, such as bioethanol production with fermentation (5), and (*ii*) through thermochemical processes, such as gasification combined with Fischer–Tropsch processing (6, 7) or pyrolysis with catalysis and upgrading (8). For the biochemical pathway, although proven technologies are available for converting sugars and grains to ethanol, BECCS using biochemical processes faces challenges such as land-use limits and food security concerns (5). In contrast, thermochemical processes that use crop residues as fuel stocks have been suggested in a number of studies as a more promising carbon mitigation option (9, 10). However, the most important obstacle in this case, at least in the near term, is the competition posed by persistently low oil prices (9, 11, 12).

This analysis focuses on an alternative pathway that relies on thermochemical conversion of coal and crop biomass to generate electricity. Specifically, mixtures of coal and crop residues are used as fuel inputs to an integrated gasification combined cycle (IGCC) system to produce electricity. Through this process, CO_2_ emissions are concentrated and ready for CCS (hereafter referred to as CBECCS to signify coal and biomass energy inputs). This pathway has multiple advantages. CBECCS produces large quantities of baseload electricity that can be easily integrated into existing power markets. It also has flexibility with respect to the coal-to-biomass ratio, carbon intensities, and processing scales. Both features are favorable for immediate deployment while contributing to commercialization in the longer term.

Here we use China as an important test case for two reasons. First, CBECCS technology offers an opportunity for China to simultaneously address its long-term climate challenges and short-term air pollution problems (13). As the largest CO_2_-emitting country, China contributed 9.6 gigatons (Gt) of energy-related CO_2_ emissions (mainly from coal) in 2015, accounting for 26.4% of total global emissions (14, 15). China also pledged in the Paris Agreement to peak its carbon emissions by 2030 or earlier, to reduce its carbon intensity by 60 to 65%, and to increase nonfossil energy to 20% of its total primary energy consumption by the same time (16). CBECCS systems can thus contribute to China’s commitment to decarbonize its energy system. Furthermore, in contrast to traditional coal-fired power plants, CBECCS systems also remove nearly all of the particulate matters (including the particulate matter with an aerodynamic diameter of less than 2.5 μm, PM_2.5_), nitrogen oxides (NO_x_), and sulfur dioxide (SO_2_) from syngas before initiating the combustion process to generate electricity (6, 17, 18). As a result, per-kilowatt-hour emissions of PM_2.5_, NO_X_, and SO_2_ from a CBECCS plant are significantly lower than those from pulverized coal (PC) power plants. Furthermore, burning crop residues (in open fields and in conjunction with residential cooking and heating) is an important source of outdoor and indoor air pollution in China at present (19, 20). By using crop residues as fuel input, CBECCS systems could avoid air pollution and health impacts associated with biomass burning, as is demonstrated below. Therefore, deploying CBECCS could bring localized, near-term air-quality cobenefits while facilitating a smooth transition toward a carbon-neutral and ultimately carbon-negative electric power system in the future.

Second, at a time when global CCS deployment appears to be faltering, China stands out as a particularly promising opportunity to advance CO_2_ capture via gasification, which is as a key component of CBECCS. Among the three CO_2_ capture approaches—precombustion (e.g., via gasification), postcombustion, and oxy-combustion capture—only postcombustion is advancing, notably by the Petra Nova coal CCS retrofit project in Texas that came online in 2017 (21). The other two approaches have not advanced much to date. However, while many planned or initiated IGCC-CCS projects in other places have been canceled, the GreenGen IGCC demonstration project in China is an exception; phase I has been under successful operation for 7 y, since 2012 (22). Phase II is scheduled to come online in the 2020s, with the goal of ultimately integrating key technologies including IGCC and CO_2_ capture, utilization, and storage (22). Therefore, China and its GreenGen project could offer a promising near-term opportunity to advance the technology of coal and biomass gasification with CCS.

This study adopts a holistic approach to evaluate the cost performance, carbon mitigation potential, and air-quality benefits of deployment of CBECCS systems using crop residues in China. Based on simulations of the CBECCS systems using Aspen Plus (11, 23), energy flow and carbon footprints are evaluated for the entire thermochemical conversion processes. We assess then their cost competitiveness compared with supercritical PC (SC-PC) plants under various carbon prices. In addition, we quantify the air-quality cobenefits of deploying 150-GW CBECCS systems in mainland China (based on projected scale of future coal additions), which utilizes around 24.3% of available crop residues (*SI Appendix*, Table S8).

We highlight three findings. First, with a mass fraction of crop residues greater than 35% in the coal–biomass fuel mixture, CBECCS systems could generate electricity with net-zero life-cycle emissions of GHGs (in CO_2_-eqivalent). Second, when the carbon price reaches $52.0 per ton of CO_2_, net-zero GHG CBECCS systems become economically competitive compared with traditional PC power plants, with a levelized cost for electricity (LCOE) of roughly 9.2 US cents per kilowatt hour. The cost competitiveness of the CBECCS systems is also affected strongly by the price of biomass. Finally, deployment of CBECCS systems can significantly reduce air pollutant emissions and improve air quality. For instance, in the highly polluted North China region, the potential reduction in air pollutants (SO_2_, NO_X_, and primary PM_2.5_) from deployment of ∼24.3 GW of CBECCS systems could lead to a 6.8% reduction in annual average PM_2.5_ concentration in 2015. This measure alone could contribute to more than 27% of the pollution-reduction target that was announced for the Beijing–Tianjin–Hebei (BTH) part of the North China region in the Action Plan on Prevention and Control of Air Pollution issued by China’s State Council. While CBECCS systems currently have relatively high costs, air pollution concerns provide an additional incentive for early deployment and may facilitate long-term cost reduction as learning progresses.

## Results

### From Coal/Biomass to Syngas and Electricity.

**Fig. 1.**
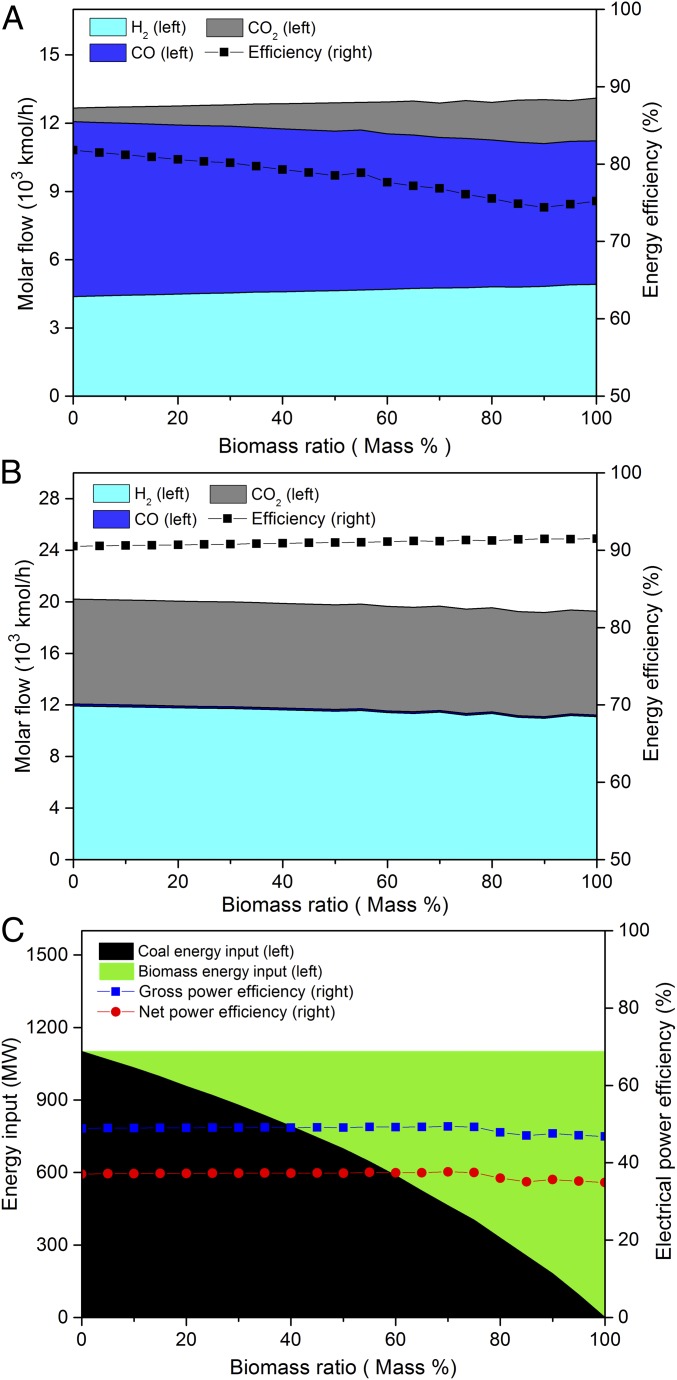
Performance of CBECCS systems with mass mixing ratios of biomass varying from 0 to 100%. (*A*) Gasification process: the composition of syngas (CO, H_2_, and CO_2_) and the associated energy conversion efficiencies (the ratio of energy output to input in lower heating value, LHV). (*B*) WGS process: the production of CO, H_2_, and CO_2_ and the associated energy conversion efficiencies. (*C*) Overall electricity generation efficiency: energy inputs from coal and biomass and the net and gross efficiencies of electricity generation in the CBECCS systems.

The CBECCS system starts with the gasification process, in which the solid feedstock of coal and biomass is converted into a gaseous fuel, that is, syngas comprised mainly of H_2_, CO, and CO_2_ (24)_._ We consider an entrained-flow gasifier (EF) that operates typically at high temperatures (1,300 to 1,500 °C), such that almost all of the coal and biomass mixture in the feedstock (more than 99.5%) is gasified (11, 23). The high-temperature gasification process is effective in tar reduction, which makes it more tolerant than conventional power plants with respect to feedstock heterogeneity (25, 26). In addition, the gasification option allows for a significant reduction in air pollutant emissions compared with direct combustion of these fuels (27). The solid-fuel feedstock is oxidized partially in the process, not only providing energy for the endothermic reactions in the gasifier that generate CO and H_2_ (Fig. 1) but also compensating for system energy losses (25, 28).

Fig. 1*A* illustrates the modeling results for EF gasification with mixing ratios of crop-residue biomass (CrB) ranging from 0 to 100%. Holding constant the total energy input from coal and crop residues, we find an increase in the CO_2_ ratio in the output gases as the biomass ratio increases. Meanwhile, the conversion efficiency decreases from ∼81.8 to 75.2% with increasing biomass ratio. Due to the relatively high moisture and volatile matter contained in biomass (*SI Appendix*, Table S6) and the greater contributions of oxygenated chemical bonds (e.g., C–O, C=O, and O–H) compared with coal, a higher biomass ratio requires additional energy in the gasification process to break these bonds. Further, at higher biomass ratios, gasification of the feedstock produces slightly higher H_2_ and lower CO in the syngas, driven by higher moisture content of biomass (25). The resulting syngas is eventually deployed in the combustion process to generate electricity.

Through the water–gas shifting (WGS) process $\left(\text{CO}+{\text{H}}_{2}\text{O}↔{\text{CO}}_{2}+{\text{H}}_{2},\Delta \text{H}\left(298\text{K}\right)=-41.2\;\text{KJ}/\text{mol}\right)$, most of the carbon content of the feedstock is converted to CO_2_. The CO_2_ concentrations produced through the WGS process increase from 4% (3.7∼4.3%) to 26% (24.9∼27.4%) (*SI Appendix*, Tables S3 and S4). As the WGS process is exothermic, the syngas from the EF gasifier outlet is precooled from around 1,300 °C to 200 °C through water quenching to facilitate reaction in the forward direction (23). Approximately 16.0% of the energy in the raw material is recovered in the form of steam from gasification and the WGS process, which can be channeled to a heat-recovery steam-generator system (HRSG) to enhance the overall efficiency of electric power generation. The WGS conversion efficiency exhibits a slightly increasing trend as a function of growing share of biomass inputs (Fig. 1*B*). This reflects the fact that additional levels of biomass lead to a lower ratio of CO in the gasification-produced syngas, reducing load requirements for the WGS reaction.

The shifted syngas consists mainly of H_2_ (35.6 to 40.1%), CO_2_ (24.9 to 27.4%), and H_2_O (31.2 to 38.2%). CO_2_ and other acid gases, including H_2_S and COS, are removed from the shifted syngas using the Rectisol method employing methanol as the working fluid (23). During the acid gas removal (AGR) process, additional energy is required for thermal regeneration of solvent and the absorption/desorption cycles of CO_2_. Roughly 6.4 to 11.6% of the gross electricity generation is consumed internally for the air separation unit to separate oxygen and for AGR to compress the CO_2_ stream up to 150 bars for utilization (e.g., to enhance oil recovery or to prepare for final sequestration). *SI Appendix*, Table S5 summarizes the feedstock composition and CO_2_ emissions per kilowatt hour assuming a rate of CO_2_ capture of around 90%.

As illustrated in Fig. 1*C*, both gross and net efficiencies for production of electricity by the CBECCS system decrease slightly with the increase of the biomass share in the feedstock. Although the addition of biomass requires less energy to prepare the feedstock and recovers more heat by the HRSG compared with coal, the high moisture content of biomass requires more oxygen in gasification and results in more CO_2_ to capture, compared with the coal-only case, CBECCS-CrB0 (Fig. 1*B* and *SI Appendix*, Tables S4 and S5). Due to the high internal energy consumption, CBECCS systems could produce electricity at a net efficiency of 32.16 to 35.70%, which is lower than that of advanced PC power plants without CO_2_ capture (∼42.7%) (22, 29).

### Direct Carbon and Life-Cycle GHG Footprints.

**Fig. 2.**
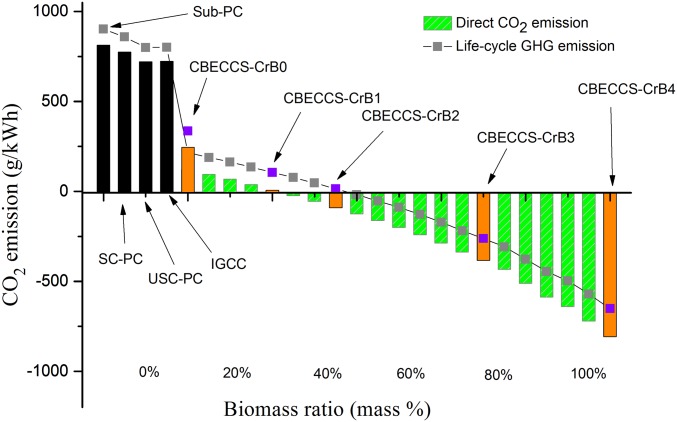
Direct emissions of CO_2_ and life-cycle emissions of GHGs from CBECCS systems, coal-fired power plants (PC), and IGCC plants without CCS. The bars represent direct CO_2_ emissions. The squares represent the life-cycle GHG emissions, expressed in total CO_2_-equivalent.

We evaluate the direct CO_2_ emissions and life-cycle emissions of GHGs (measured in CO_2_-equivalent) for CBECCS systems and compare them with those from PC and coal-fired IGCC plants in China. Direct emissions of CO_2_ occur only from the combustion of coal in power plants (the bars in Fig. 2), while the life-cycle emissions of GHGs (the squares in Fig. 2) include also the GHG emissions from the preprocessing of coal and biomass before entering the power-generation systems (*SI Appendix*, section S3) (30, 31). Biomass combustion does not contribute to CO_2_ emissions, as the carbon content in biomass is derived from the atmosphere through photosynthesis.

We consider a range of biomass ratios for the CBECCS system, including a coal-only case labeled as CBECCS-CrB0 (i.e., 0% crop residue) and four cases with 20%, 35%, 70%, and 100% biomass labeled as CBECCS-CrB1 to -CrB4, respectively (more information is given in *SI Appendix*, Table S2). Evaluating a range of biomass ratios for CBECCS is warranted because transitioning China’s coal-dominated thermal power system to increasing reliance on biomass fuels would have to occur gradually, due to (*i*) feasibility concerns, considering the time required both to establish an efficient collection system for crop residues on a large scale and to make adjustments in coal supply chains (e.g., mines and transport), and (*ii*) institutional and political reasons, to mitigate resistance from incumbent coal interest groups.

Compared with PC plants and traditional IGCC without CCS (black bars), Fig. 2 summarizes the life-cycle GHG emissions (in CO_2_-equivalent) associated with the production of 1 kWh of electricity from CBECCS systems with biomass ratios ranging from 0 to 100%. Zero-emission electricity in terms of direct CO_2_ and life-cycle GHGs is achieved at the crop-residue fractions of 20% and 35% in the fuel mix (or CBECCS-CrB1 and -CrB2), respectively. With biomass fractions higher than 35%, CBECCS systems become negative-emission electricity-generating technologies in terms of not only direct CO_2_ but also life-cycle GHGs.

The CO_2_ produced as a by-product from the CBECCS systems could be utilized and stored in depleted gas basins, employed for enhanced recovery of oil or coalbed methane, or sequestered in appropriate geological reservoirs (e.g., deep saline sedimentary formations) (11, 32–35). For the CBECCS deployment scenario (e.g., 150 GW in total) to be discussed later, annual CO_2_ potentially required for sequestration amounts to 129 megatons (Mt), 164 Mt, 169 Mt, 169 Mt, 94 Mt, and 77 Mt, respectively, for the six regions of China’s mainland, namely North China, Northeast, East China, South Central China, Southwest, and Northwest, which is negligible compared with the available onshore geological repositories in China (i.e., less than 0.036% of total repositories) (34, 36–38).

### Levelized and Marginal Costs of Negative Carbon Electricity.

We evaluate the LCOE for the five different biomass-mixing ratios (i.e., 0% in CBECCS-CrB0 to 100% in -CrB4) and compare them with results for SC-PC and IGCC plants. Without a carbon price, the LCOE increases from 8.78 US cents per kilowatt hour for CBECCS-CrB0 to 9.98 US cents per kilowatt hour for CBECCS-CrB4. The SC-PC power plants have the lowest LCOE, at 4.67 US cents per kilowatt hour, consistent with the bus-bar coal electricity prices currently available to grid companies in China (39). Due to its low LCOE, coal has been the dominant fuel in the electric power system in China, increasing from 1,114 TWh in 2000 to 4,284 TWh in 2015 (40). The results indicate that in the absence of carbon taxes or regulation to restrict CO_2_ emissions, deployment of CBECCS plants from an economic perspective would not be currently attractive in China.

**Fig. 3.**
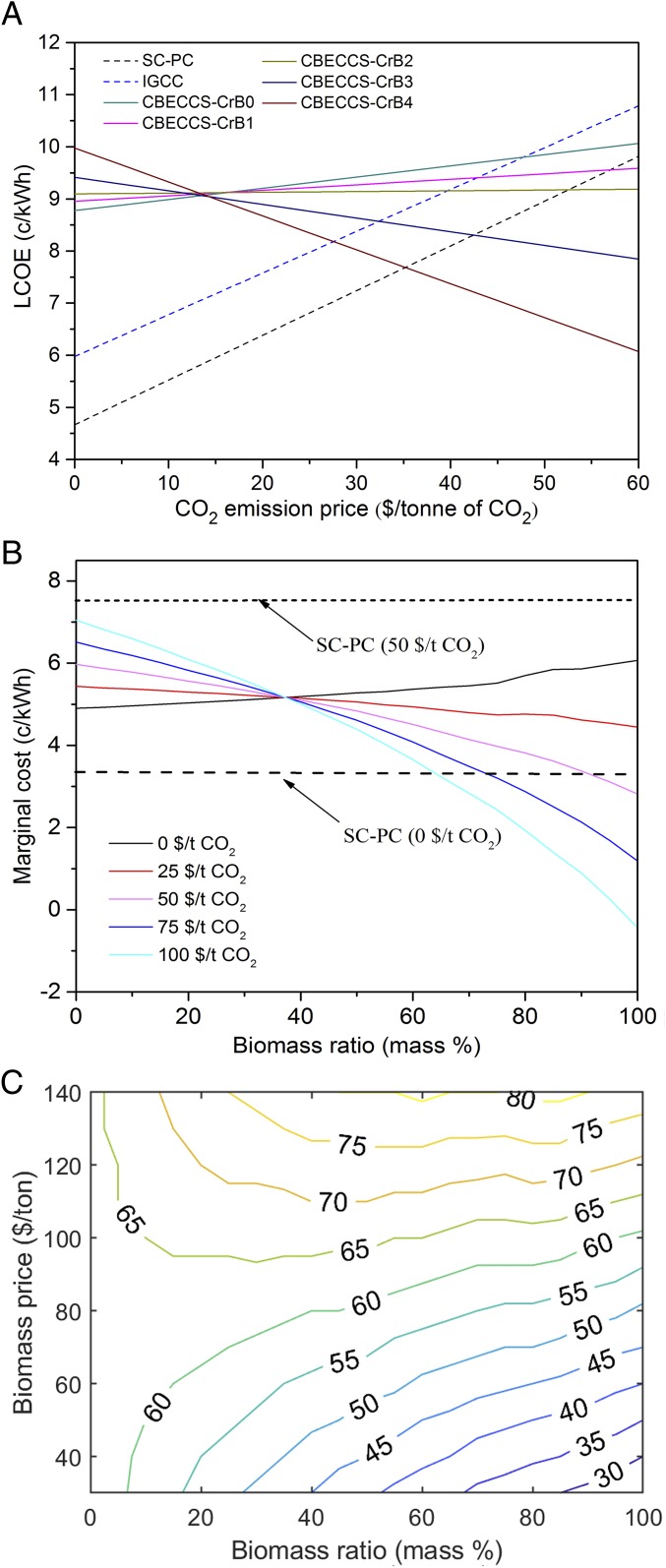
Economic analysis of electricity generation of CBECCS systems. (*A*) LCOE for coal-fired power (PC) plants, IGCC plants, and CBECCS systems, with a carbon price from 0 to $60 per ton of CO_2_. (*B*) Marginal costs of electricity generation as a function of mass ratios of biomass and carbon prices. The marginal cost of CBECCS-CrB4 becomes negative with a carbon price higher than $100 per ton of CO_2_. (*C*) Break-even carbon price to make CBECCS systems cost-competitive with PC plants, as a function of prices and mass mixing ratios of biomass. The colored lines are isoquants with the same break-even carbon prices. The mass mixing ratio of biomass in the fuel stock varies from 0 to 100%.

Fig. 3*A* illustrates the influence of carbon prices on the LCOE of CBECCS systems. CBECCS-CrB1 is associated with zero direct carbon emissions and its LCOE is independent therefore of carbon price. For the plant configurations with positive carbon emissions, specifically SC-PC, IGCC, and CBECCS-CrB0, the LCOE increases with rising carbon prices. In contrast, for the plant configurations with negative direct emissions (i.e., CBECSS-CrB2 to -CrB4) the LCOE declines with increasing carbon prices. Moreover, the slopes become steeper with higher mixing ratios of biomass (e.g., from CBECCS-CrB2 to -CrB4), suggesting that higher carbon prices could effectively encourage CBECCS systems to transition toward higher biomass ratios as input fuel.

On the basis of LCOE, we find the break-even carbon prices to be $42.0 and $52.0 per ton of CO_2,_ to make net-zero GHG-emission CBECCS (i.e., the CBECCS-CrB2 configuration) cost-competitive compared with coal-based IGCC and SC-PC plants, respectively.

The marginal cost of CBECCS systems depends on fuel costs, operational and maintenance (O&M) costs, and the price of carbon. With a carbon price of $100 per ton, a CBECCS-CrB4 system with 100% biomass would produce carbon-negative electricity at a negative marginal cost. (Fig. 3*B*). Notably, with such a high price on carbon and high relative ratio of biomass, the short-run marginal cost of CBECCS could be lower even than that for renewable electricity (i.e., essentially zero). This implies that under a merit-order dispatch approach based on marginal costs, CBECCS, as a dispatchable generation source, could potentially have the highest priority and be dispatched first among all generation sources. This could guarantee a high capacity factor for CBECCS units, compensating for related capital and fuel costs. However, dispatch decisions in China do not follow economic merit-order procedures at present. Instead, the government assigns fixed operating hours to each class of power plants (41). In the near term, since CBECCS also uses coal, it is possible that CBECCS power plants could follow existing rules and practices for coal power plants, with guaranteed operating hours. In the long run, as China continues the ongoing market-oriented power sector reform (41, 42), a transition toward merit-order dispatch may better reflect the economics and give priority to negative-cost electricity generated from CBECCS with a high carbon price and biomass ratio.

The biomass price could be influenced, however, by a variety of factors, including collection radius and costs for transportation and storage (more discussion in *SI Appendix*, section S2.2). We explore here, at various biomass ratios, how the prices for biomass would affect the break-even price for carbon, that is, the level at which CBECCS-CrB2 becomes cost-competitive compared with SC-PC plants (Fig. 3*C*). With no biomass fuel inputs (i.e., CBECCS-CrB0), the break-even carbon price is around $63 per ton of CO_2_, independent of biomass price. As shown in Fig. 3*C*, with a biomass price lower than $80 per ton, the break-even price for carbon decreases as a function of biomass mixing ratios. This indicates that adding biomass to the feedstock leads to a lower cost for reducing CO_2_ emissions. For example, at the current crop-residue price of $50 per ton, the break-even price for carbon decreases from $63 per ton for CBECCS-CrB0 that uses only coal to $52 per ton for CBECCS-CrB2 with a biomass mixing ratio of 35%. However, if the biomass price exceeds $80 per ton, the break-even carbon price would increase with an increasing biomass ratio.

### Carbon Mitigation and Air-Quality Cobenefits.

**Fig. 4.**
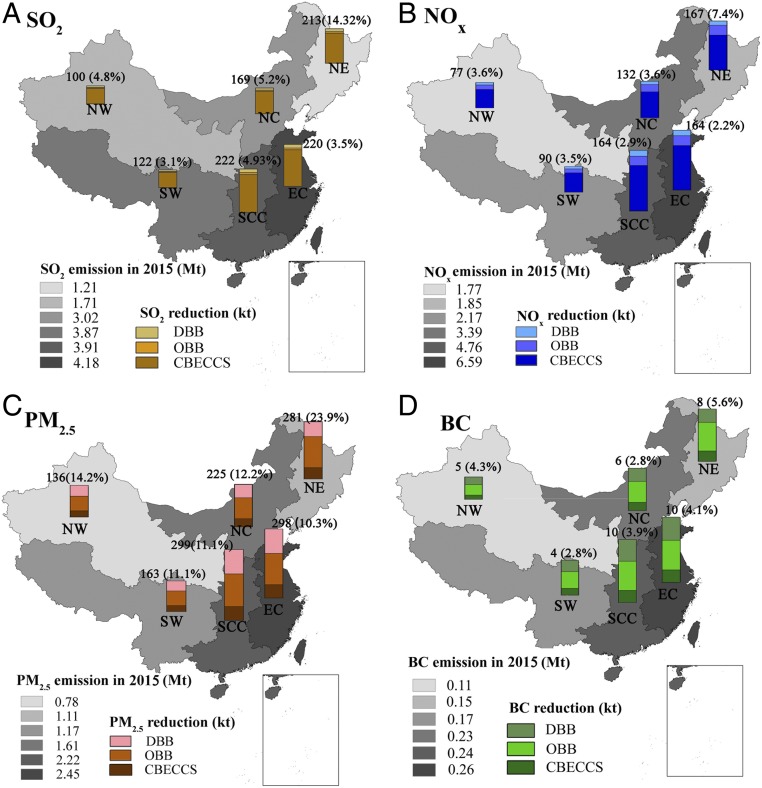
Reductions in annual total emissions of air pollutants achieved by the CBECCS-CrB2 deployment scenario with a mass mixing ratio of biomass of 35%: (*A*) SO_2_, (*B*) NO_X_, (*C*) PM_2.5_, and (*D*) BC. We present results for six regions in mainland China: North China (NC), Northeast (NE), East China (EC), South Central China (SCC), Southwest (SW), and Northwest (NW). The bars represent the emission reductions from displacing coal-fired power (PC) plants with CBECCS systems and from avoided OBB and DBB.

Compared with PC plants or direct biomass burning, electricity generation from CBECCS systems has lower carbon and air pollutant emissions. To shed light on potential carbon and air-quality cobenefits of CBECCS deployment, we designed a counterfactual scenario for 2015 in which CBECCS are deployed to displace recently built PC plants in China that are primarily supercritical and ultra-supercritical units. In a carbon-constrained world, these young coal plants may need to retire early by midcentury, the likely time horizon when CBECCS can start to play a more important role. Specifically, we develop a scenario where a total of 150 GW of net-zero GHG emission CBECCS units (CBECCS-CrB2) are deployed, based on the scale of projected coal additions by the International Energy Agency (43). Specifically, we assume ∼24.3% of crop residues available in mainland China are utilized as input fuel, which can thus support the deployment and operation of 366 net-zero GHG emission CBECCS units (i.e., CBECCS-CrB2), each with a capacity of 410 MW (44). With a capacity factor of 80% for CBECCS, this scenario could replace 1,051 TWh of electricity generated from coal-fired power plants, equivalent to 18.1% of total electricity generated in China in 2015 (40). Displacing this amount of coal-fired electricity produced by ultra- or supercritical units could reduce annual emissions of CO_2_ by as much as 0.88 Gt, equivalent to 9.3% of total carbon emissions in China (9.6 Gt) in 2015 (Fig. 4).

Besides its potential contribution to carbon abatement, deploying CBECCS systems to displace coal power generation could also lead to a reduction in conventional air pollutant emissions, contributing thus to China’s near-term targets for air pollution control (13). Deployment of CBECCS reduces air pollution in two ways: (*i*) displacing coal-fired power generation and associated air pollution and (*ii*) avoiding biomass that might otherwise be consumed in more polluting ways such as open biomass burning (OBB) and domestic biomass burning (DBB). As a traditional practice for Chinese farmers, 17 to 25.6% of crop residues are burned in the field (10, 27, 28). OBB is thus a major source of air pollution, especially direct emissions of particulate matters including black carbon (BC). Our results indicate that the deployment scenario envisaged here could contribute to significant reductions in primary air pollutants, including NO_X_, SO_2_, PM_2.5_, and BC in all regions, especially in North and East China, where smog episodes with high levels of air pollution occur frequently (*SI Appendix*, Fig. S5).

For example, deploying 24.3-GW CBECCS-CrB2 systems in North China could reduce emissions of SO_2_ by 169.3 kilotons (kt), NO_X_ by 132.4 kt, primary PM_2.5_ by 225.2 kt, and BC by 8.8 kt, equivalent respectively to 5.2%, 3.6%, 12.2%, and 3.8% of total regional emissions in 2015. Based on simulation of air quality using Weather Research and Forecasting–Community Multi-scale Air Quality (45), the scale of reductions in air pollutant emissions achieved by the CEBCCS-CrB2 deployment scenario could decrease the annual average concentration of PM_2.5_ by 6.8% in the North China region (19). To put this in context, China set a target of decreasing the annual average concentration of PM_2.5_ by 25% in the BTH region of North China from 2012 to 2017, as announced in the Action Plan on Prevention and Control of Air Pollution issued by China’s State Council. Our CEBCCS-CrB2 scenario could achieve more than 27.2% of this PM_2.5_ reduction goal. Given that the cost to the GDP for the implementation of the action plan for BTH region in 2017 was estimated at approximately $61 billion, the potential savings that CBECCS deployment may offer in the costs of air pollution control could be significant (46, 47). The percent decrease in PM_2.5_ concentrations is expected to be even greater in winter, when residential biomass burning contributes significantly to severe air pollution episodes in China (48). In addition, since BC emissions contribute to both air pollution and local climate forcing (as a warming aerosol), decreasing BC emissions via CBECCS deployment would lead to reduced pollution as well as reduced warming.

## Discussion

### Pathways for Deploying CBECCS in China.

Deploying CBECCS systems that use crop residues as biomass input represents a win–win strategy to curb air pollution and carbon emissions in China (49, 50). There can be four major benefits of CBECCS deployment in China: (*i*) CBECCS can ultimately achieve negative GHG emission as the biomass ratio increases; (*ii*) OBB/DBB and associated air pollution could be avoided by utilizing biomass as fuel inputs to the CBECCS system; (*iii*) farmers can gain additional compensation from selling crop residue biomass, which can benefit rural economic development; and (*iv*) compared with other countries or regions such as the United States and the European Union, the capital and operating costs for the CBECCS system are likely to be much lower in China, providing a lower-cost opportunity for deployment (22, 51). While our analysis focuses on China, many countries in the developing world, such as Brazil and India, also face the challenge of addressing climate change as well as serious air pollution from biomass burning. The CBECCS roadmap in China hence also has reference value for the developing world to harness the cobenefits of mitigating both air pollution and CO_2_ emissions.

To achieve a greater role of CBECCS in China’s long-term decarbonization strategy, near-term deployment could focus on a few provinces that have an abundant supply of biomass and opportunities for CO_2_ sequestration on the one hand and that are also under pressure to curb local coal use and reduce air pollution on the other hand. As illustrated in *SI Appendix*, Fig. S4 and Table S8, production of crop residues in China is concentrated particularly in two grain-producing areas, namely the Huang-Huai-Hai region and the Northeast Plain. The top five areas in China for crop density are located in 10 provinces that also have large local electricity demand and suffer from serious local air pollution (*SI Appendix*, Table S17) (40). In addition, Huabei and Yuwan basins, covering Hebei, Hena, Shandong, and Anhui provinces, have abundant sequestration capacities for CO_2_, estimated at 264 Gt and 186 Gt, respectively (*SI Appendix*, Table S18). Judging on basis of these criteria, we suggest therefore that four provinces—Shandong, Henan, Hebei, and Anhui—could be candidates for early demonstration and initial deployment of CBECCS. These provinces have a sufficient supply of crop residues, abundant CO_2_ sequestration capacities, large existing local thermal generation fleets, and significant local emissions of carbon and air pollutants (Fig. 4 and *SI Appendix*, Fig. S5 and Table S8). Deploying CBECCS systems in these provinces could utilize local crop residues and curb air pollution and at the same time increase green electricity generation.

To deploy CBECCS technology on a large scale in China will require overcoming a number of barriers, including managing the risks and uncertainties associated with relevant technologies, biomass collection, and carbon policy. First, CBECCS systems depend on a complex combination of advanced technologies including EF gasification, WGS conversion, CCS, and hydrogen combustion in gas turbines. Although IGCC, a key component of CBECCS, is a mature technology in the United States and Europe, its application in China is still at the demonstration phase. Research and development programs and demonstration projects are required for China to master the core technologies and to gain experience to avoid technical risks (50).

Second, to ensure a reliable supply of bioenergy at a large scale, a point-to-point biomass collection network must be established in agricultural and/or forested areas to improve collection efficiency (6). Centrally mechanized crop harvests could not only lower the costs for collecting crop residues but also contribute to an increase in agricultural productivity (52). As the supply of biomass fluctuates with season, storage facilities will also be required to guarantee a stable and reliable supply of fuel feedstock for CBECCS systems. Some pretreatment measures, such as pelletization and torrefaction, could be applied to reduce storage space and risks (53). In addition, switching from crop residues to larger-scale and more reliable substitutes, such as forest biomass, could contribute to a more stable supply (54).

Third, despite significantly lower emissions of CO_2_ and pollutants, the capital and fixed O&M costs for CBECCS systems are 102.17% and 117.94% higher than SC-PC power plants, respectively (22). Without a price on emissions, especially CO_2_, it is uneconomical for CBECCS to compete with conventional coal-fired power plants at present and to realize the associated carbon and environmental cobenefits. In China, a national carbon market, starting with the electricity sector, was announced in December 2017 and is now planned to come fully online in 2020. It will introduce a price on carbon emissions that should favor low-carbon technologies such as CBECCS (6). Additional incentives would be required to reach the critical break-even point (around $52.0 per ton of CO_2_) to effectively facilitate large-scale application of CBECCS.

### The Role of CBECCS as Part of a Broad CCS Roadmap for China.

In the broad context of designing China’s CCS strategy, besides gasification-based precombustion CO_2_ capture technology as the focus of this study, postcombustion capture has also been viewed as a promising technology choice, especially as an option to retrofit existing coal-fired plants (43). Successful commercial-scale demonstrations have already been realized in China and elsewhere (22). However, retrofitting existing coal power plants entails logistic challenges, such as whether there is a CO_2_ storage site in the proximity and whether there is adequate on-site space to add postcombustion facilities. In comparison, since the pace of new coal plant additions in China is projected to slow down in the coming decades, the market for CBECCS via gasification is likely to involve replacing coal power plants that will be retired by midcentury or later (55). For consistency with such a time horizon, this study compares the economic and environmental implications of CBECCS with most advanced coal units at present, that is, supercritical and ultra-supercritical coal units.

To inform China’s long-term roadmap for CCS and how the CBECCS market should be divided between gasification and postcombustion approaches, the policymakers and investors need to compare the option of constructing new CBECCS plants using precombustion capture technology, with the strategy of retrofitting or constructing coal power plants using biomass cofiring and postcombustion capture technology. Answering these questions requires future research on plant-based evaluation of existing coal plants for their space and water availability to add and operate postcombustion capture facilities, and for the associated changes in costs and efficiency (43, 56, 57). Then the option of retrofitting existing plants can be compared with the option of building new plants that use gasification or postcombustion approaches.

Although a quantitative comparison goes beyond the scope of the present study, qualitatively CBECCS with gasification has a number of advantages over postcombustion technology. Most importantly, although retrofitting conventional coal units with postcombustion CCS can certainly lower carbon emissions, it is constrained by a technical limit for the biomass cofiring ratio, which consequently limits the carbon mitigation potential. At present, the biomass share in biomass/coal cofired plants is usually below 5% and rarely exceeds 10% on a continuous basis, although 20% cofiring is technically feasible (58). In contrast, CBECCS technology can operate not only at high biomass ratios but can achieve zero life-cycle CO_2_ emissions with a biomass ratio as low as 35%. Therefore, given the ultimate need for negative carbon emissions to address the climate challenge, CBECCS via gasification provides a more promising opportunity to gradually increase the biomass ratio, thus laying the foundation for a complete shift away from fossil energy and for negative-carbon electricity in the long run.

## Methods

**Fig. 5.**
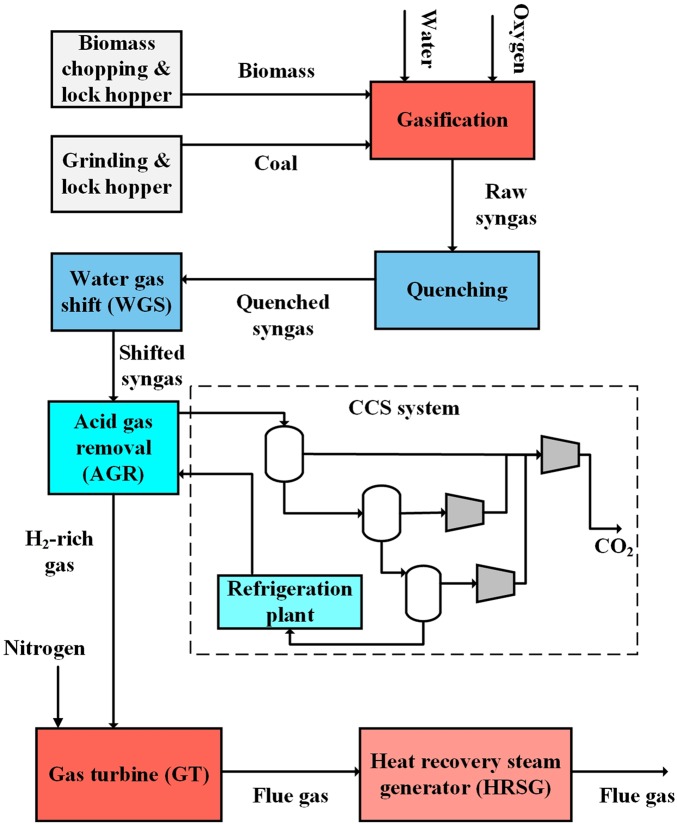
Process flow diagram of the CBECCS system for electricity generation using IGCC technology with CCS.

The CBECCS system was simulated using Apsen Plus software with assumptions for currently available, state-of-the-art processes. Twenty mixing ratios of the crop residue were simulated with mass and energy flows balanced at each step and validated with existing literature (11, 23). A flow diagram of the CBECCS system for electricity generation is illustrated in Fig. 5, with detailed information on model parameters and inputs and outputs of materials and energy summarized in *SI Appendix*, Tables S1–S6.

Life-cycle emissions of GHGs from coal and biomass in the CBECCS systems are evaluated using the standard ISO (International Organization for Standardization) model with consideration of both operational emissions and upstream emissions associated with production, processing, and transportation of coal and biomass. Detailed data are summarized in *SI Appendix*, section S3 (30, 59). Cobenefits in air pollution mitigation were evaluated for primary air pollutants including SO_2_, NO_X_, PM_2.5_, and BC. The emission factors of these species for CBECCS systems, coal-fired power plants, OBB, and DBB were adopted from an emission inventory for China’s air pollution developed by Tsinghua University and documented in existing literature (*SI Appendix*, Tables S15 and S16) (10, 51, 60–62).

The LCOEs for CBECCS, coal-fired IGCC, and traditional power plants were evaluated using a cash-flow financial model developed for this analysis. The economic parameters for Nth of a kind CBECCS-CrB0 were taken from a case study of the GreenGen (IGCC) Project in Tianjin, China, presented by the Asian Development Bank (22). As the fraction of biomass increases from 0% in CBECCS-CrB0 to 100% in -CrB4, we assume that the overnight capital investment will increase by 10% and fixed O&M costs by 30% (10, 63). The economic parameters for different power-generating units and cash-flow models are described in *SI Appendix*, Table S14. Prices for coal in China were taken as $80 per ton based on the average Bohai-Rim Steam-Coal Price between 2017 and 2018 (64), and biomass prices were estimated as a function of transport distance and biomass density with parameters calibrated using existing literature (*SI Appendix*, section S2.2) (65). A sensitivity analysis for the LCOE in terms of capital costs, discount rate, and fuel prices is illustrated in *SI Appendix*, Fig. S3.

The present analysis also quantified the influence on the LCOE of different electricity-generating technologies of carbon taxes ranging from 0 to $60 per ton of CO_2_. The reduction costs for CO_2_ emissions $\left(C^{CO_{2}}\right)$ using CBECCS systems compared with SC-PC power plants were quantified using the following equation:

$$C^{CO_{2}}=\frac{P_{kWh}^{CBECCS}-P_{kWh}^{PC}}{E_{CO2}^{CBECCS}-E_{CO2}^{PC}},$$

where $P_{kWh}^{CBECCS}$ and $P_{kWh}^{PC}$ refer to the LCOE realized, respectively, by a CBECCS system and a SC-PC plant and $E_{CO2}^{CBECCS}$ and $E_{CO2}^{PC}$ indicate emissions of CO_2_ associated with production of 1 kWh of electricity using CBECCS and SC-PC power plants, respectively.

## Supplementary Material

Supplementary File
