# Supplementary material for: Gasification of coal and biomass as a net carbon-negative power source for environment-friendly electricity generation in China
Source: Proc Natl Acad Sci U S A. 2019 Apr 8;116(17):8206–13. doi: 10.1073/pnas.1812239116 (PMC6486764; doi:10.1073/pnas.1812239116)
Supplement: Supplementary File [file pnas.1812239116.sapp.pdf]

Supporting Information for the paper "Gasification of coal and biomass: a net carbon-negative power source for environment-friendly electricity generation in China"

Xi Lu[a*], Liang Cao[a,b], Haikun Wang[c], Wei Peng[d], Jia Xing[a], Shuxiao Wang[a], Siyi Cai[a], Bo Shen[e], Qing Yang[f,g], Chris P. Nielsen[g], Michael B. McElroy[g,h*]

a. School of Environment and State Key Joint Laboratory of Environment Simulation and Pollution Control, Tsinghua University, Beijing 10084, P.R. China

b. School of Chemical Engineering, The University of Queensland, St. Lucia, Brisbane, QLD 4072, Australia

c. State Key Laboratory of Pollution Control and Resource Reuse, School of the Environment, Nanjing University, Nanjing 210023, P.R. China

d. School of International Affairs and Department of Civil and Environmental Engineering, Pennsylvania State University, University Park, Pennsylvania 16802

e. China Energy Group, International Energy Analysis Department, Lawrence Berkeley National Laboratory, 1 Cyclotron Road, Berkeley, CA 94720 USA

f. Department of New Energy Science and Engineering, School of Energy and Power Engineering, Huazhong University of Science and Technology, Wuhan 430074, PR China

g. John A. Paulson School of Engineering and Applied Sciences, Harvard University, Cambridge, MA 02138, USA.

h. Department of Earth and Planetary Sciences, Harvard University, Cambridge, MA, 02138, USA.

*Corresponding authors:

Michael B. McElroy (mbm@seas.harvard.edu), Xi Lu (xilu@tsinghua.edu.cn)

www.pnas.org/cgi/doi/10.1073/pnas.1812239116

The following document is the supporting information (SI) for the paper "Gasification of coal and biomass: a net carbon-negative power source for environmental friendly electricity generation in China". Section S1 summarizes modeling parameters and results for the processes of gasification, CO_2 capture and electricity generation using Aspen Plus software. Section S2 describes the method for evaluating the costs of collection, processing and transportation of crop-residue biomass in China. Section S3 discusses life-cycle GHG emissions measured in CO_2-equivalents (CO_2-eq) for coal and crop-residue biomass during pre-treatment and thermal conversion in CBECCS (Coal-Biomass Energy with Carbon Capture and Storage) systems. A cash flow model and the relevant economic parameters to calculate levelized costs of electricity (LCOE) using different technologies are presented in Section S4. The methodology to quantify the potential reductions in air pollutant emissions through the CBECCS systems is included in Section S5. To identify provinces for prioritized deployment of CBECCS systems in China, the last section (Section S6) compares the top 10 provinces in terms of the availability of crop residue supply, and their local emissions of CO_2 and air pollutants.

S1 CBECCS System and Aspen Plus Modeling

This study explores a new opportunity in China to use a mixture of coal and crop residues as input fuels for a CBECCS system, an integrated gasification combined cycle (IGCC) facility with carbon capture and storage (CCS), to produce electricity. The general diagram of a CBECCS system is presented in Fig. 5 in the main text, and the

design parameters for individual modules in the Aspen Plus simulation for CBECCS systems are listed in Table S1.

The solid feedstocks of coal and biomass are first milled and then gasified with oxygen and steam. The resulting syngas, mainly H_2 and CO, is further converted to hydrogen through the Water Gas Shift (WGS) process. The Acid Gas Removal (AGR) process is adopted to remove the CO_2 and other acid gases from the syngas. After this process, the syngas with approximately 90% hydrogen by volume is channeled to a gas turbine for electric power generation. The high-concentration of CO_2, as a by-product, can be either stored in suitable geological fields, utilized for Enhanced Oil Recovery (EOR) or potentially combined with hydrogen to produce a liquid fuel such as methanol (1).

The CBECCS system is modelled and simulated using the Aspen Plus platform (2-4), which is widely applied to simulate real plant operations. Aspen Plus software has the capacity to treat the engineering relationships including mass and energy balances, phase and chemical equilibria, as well as reaction kinetics. The key indicators of plant performance used in the Aspen Plus simulation are derived from existing literature (2, 4). The modeling results are summarized in Tables S2-5 and Fig. 1. IGCC technology has been deployed at the Tianjin GreenGen power plant in China to continuously produce electricity since 2012 (5). However, estimates of CO_2 storage capacity potential for China remain highly uncertain to date because of a lack of consistent evaluation models and a standardized assessment methodology, as well as limited data regarding the subsurface geological properties (5, 6).

S1.1 Gasification Process

A representative steam coal (Inner Mongolia bituminous) and crop-residue biomass (wheat straw) in China are selected for this analysis (see in Table S6). The compositions of Inner Mongolia bituminous and wheat straw are summarized in Fig. S1 and compared to other types of coal and crop residues in China (4, 7). Raw coal and biomass feedstocks need to be crushed in a mill before feeding into the gasifier through a pressurized lock hopper system. The energy required for preparation of feedstocks of coal and biomass is presented in Table S1 (4).

Solid feedstocks are converted into gaseous mixture by a gasification process in which coal and biomass are primarily decomposed into CO, H_2, H_2O and CO_2. There are three typical gasification reactors, namely moving-bed, fluidized-bed and entrained-flow (EF) reactors (8). This study examines particularly a water-and oxygen-blown, EF gasifier operating at 40 bar to emulate an operational Siemens gasifier (2). We choose EF gasification for a number of reasons: (1) this technology is commercially available; (2) it can realize large-scale and high-efficiency syngas production, with feedstocks converted almost completely to gases (more than 99% carbon conversion) within a short time due to the relatively high operational temperatures (1200-1500°C) (9); (3) it can tolerate heterogeneous coal/biomass fuel mixes (10-12) ; (4) due to its high gasification temperature, by-products such as other hydrocarbons and tars normally produced in lower-temperature gasification, can be avoided almost entirely; and (5) the gasifier can also be easily cleaned using conventional tar cleaning technologies (11, 12). Nearly pure oxygen is utilized in the gasification process to avoid introducing nitrogen into the syngas, ensuring a high concentration of CO_2. The Aspen Plus Gibbs reactor,

RGibbs, is used to simulate gasification of the coal-biomass mixture and combustion of H_2. The RGibbs module seeks the chemical equilibrium with minimum Gibbs free energy. Before feeding the coal-biomass into the RGibbs block, the fuels are decomposed into elemental forms through the RYield module based on their component yield specifications (13).

The total heat value of coal and biomass mixtures are assumed to be similar among scenarios in the present analysis. Table S1 summarizes the key design parameters and assumptions for the processes of gasification. The main chemical reactions taking place in the gasifier can be expressed as follows:

$$\mathrm{C} + \mathrm{O}_2 \rightarrow CO_2;\ \ \Delta = -394\ \ kJ/mol \tag{1}$$

$$2\mathrm{C} + \mathrm{O}_2 \rightarrow 2CO;\ \ \Delta = -221\ \ kJ/mol \tag{2}$$

$$\mathrm{C} + \mathrm{H}_2O \rightarrow CO + H_2;\ \ \Delta = 131\ \ kJ/mol \tag{3}$$

$$\mathrm{C} + CO_2 \rightarrow 2CO;\ \ \Delta = 173 kJ/mol \tag{4}$$

$$\mathrm{CO} + H_2O \rightarrow CO_2 + H_2;\ \ \Delta = -41\ \ kJ/mol \tag{5}$$

Of these chemical reactions, equations (1) and (2) represent strongly exothermic reactions, which provide the energy required for the gasification process. Equations (3) and (4) denote the main chemical reactions contributing to the production of syngas in the gasifier, which are strongly endothermic. Equation (5) represents a slightly exothermic reaction, converting CO and H_2O to CO_2 and H_2, which serves as the main reaction in the next step of the Water Gas Shift (WGS).

S1.2 WGS Process

The raw syngas produced from gasification is further processed through the WGS

to enrich the contents of H_2 and CO_2. The raw syngas is cooled to around 200°C by water quenching in order to facilitate the exothermic reaction of Equation (5) (4, 7). The reaction in the WGS converts the CO and H_2O in syngas to H_2 and CO_2. To maintain a high conversion efficiency (>95%) from CO to CO_2, the H_2O/CO ratio in the WGS is adjusted to a minimum of 2 (2). The reaction temperature is controlled within 250℃ and 300℃ to ensure the exothermic conversion proceeds forward to produce CO_2 and H_2. The heat is recovered and channeled for electric power generation in a Heat Recovery Steam Generator system (HRSG).

S1.3 Acid Gases Removal

A two-stage Rectisol® process is employed for acid gases removal (AGR). Around 80% of the CO_2 can be captured, and virtually all the air pollutants including sulfur dioxide (SO_2) and carbonyl sulfide (COS), and Hydrogen sulfide (H_2S) can be removed (2, 4). The syngas from the WGS process is first cooled through heat exchange with the lower-temperature and H_2-rich gas treated previously by the AGR unit, and then flows into two-stage absorbers where H_2S and COS are first preferentially removed using a methanol solvent. The sulfur-free syngas passes through the second absorber where CO_2 is separated using a chilled methanol solvent. A portion of the CO_2-loaded solvent is chilled and sent back to the first absorber, and the rest is pumped to a series of flash drums for CO_2 regeneration. Through different levels of decompression, the CO_2 stream is passed from high-pressure (HP), medium-pressure (MP) and low-pressure (LP) flash drums and the remaining methanol solvent is first chilled and then sent back to the absorbers. Similarly, the sulfur-rich solvent from the first absorber is heated through a

series of flash drums to regenerate the H_2S and COS, which can be sent to a Claus plant for further processing to produce sulfur (14-17). The final hydrogen concentration in the syngas treated by AGR is around 90% by volume in a dry gaseous stream.

The CO_2 streams regenerated from the flash drums are compressed to a supercritical condition at 150 bar using a multiple-stage, intercooled compressor. Then the compressed CO_2 is transported and injected into suitable saline formations or oil fields for sequestration (1). The major factors that influence the energy consumption during the CO_2 capture process include recycled use of the chilled methanol solvent and the compression of CO_2 for storage. The heat duty of pre-combustion CO_2 capture ranges from 0.7 to 1.2 MJ/kg CO_2, much lower than that of post-combustion capture (3-4 MJ/kg CO_2) (1, 2).

S1.4 Electricity Generation

In the CBECCS system, electricity is generated by a gas turbine combined with the HRSG. The hydrogen-rich gas from the AGR unit is entrained into an M701G2, a state-of-the-art Mitsubishi gas turbine with a maximum output of 370 MW and a centrifugal compressor pressure ratio of 21:1 (2). The isentropic efficiency of the gas turbine is set at 90% (2, 4). Before combustion, the hydrogen is diluted with water steam and nitrogen from the ASU unit to control the hydrogen combustion temperature. The combustion system provides around 235% excess air and limits the flame temperature of the combustor to 1400 ℃ for the gas turbine (2). The stoichiometric flame temperature (SFT) in the combustor is maintained at approximately 2300 K (~2030℃) in order not to exceed NO_x emission limits without end-of-pipe de-nitrification systems

(18). The exhaust gas from the turbine is sent to the HRSG with a capacity of 170 MW for additional generation of electricity. The isentropic efficiency of the steam turbine of the HRSG is set at 85% (2, 4). While a considerable portion of the gross electricity generated by the gas turbine and HRSG (about 23.9-24.2%) must be used for onsite ancillary functions, this leaves more than 75% to be delivered into the electric power grid.

S2 Evaluation of the potential and cost for crop-residue biomass in China

To evaluate the potential of crop residues, we first determine the amount of biomass produced annually and estimate then the amount that could be feasibly collected and utilized to produce electricity. Total cost of the crop-residue biomass supply is evaluated by accounting for separate costs of collection, processing and transportation. The 2015 exchange rate of 6.22 is adopted to convert between US dollars and RMB, and all costs are expressed in the US $ value of 2015. The biomass distribution data are collected from the National Bureau of Statistics of China (NBSC) and existing published reports (19-21).

S2.1 Potential for crop residues

Crop residue refers to the biomass left over after harvesting and processing of crops such as corn, rice and wheat, of which the availability was estimated on the basis of agricultural product yield (22). Following the methods reported by earlier studies (23-25), we evaluated the residue production for each province in China based on the residue to grain index (R/G index). Using the crop production data derived from the NBSC in 2016 (26), we estimated the total dry-basis crop residue production rate at 930

million tonnes per year in China, of which approximately 465-651 million tonnes per year are assumed to be sustainably recoverable for energy use, with an energy value of 6.89-9.65 EJ/year (27-29). It is noteworthy that the production of crop residues increased at annual rate of 1.75% between 2006 and 2016 (26), reflecting the growth of agricultural production in China driven by the expanding population together with changes in dietary habits as living conditions improved. High production of crop residues occurs in multiple regions of China, including the east, north, northeast and south. In particular, the top three provinces in terms of crop-residue biomass potential are Henan, Shandong, and Heilongjiang.

Climate-change may impact future agricultural production and thus potential residue supplies. On one hand, agricultural production may benefit from the fertilizing effect of elevated CO_2 (30). Additionally, Wang et al. reported that the increase in temperature in late fall may promote later sowing of winter wheat and later harvesting of maize, resulting in an overall 4-6% increase in total grain yield of the wheat-maize cropping system of the North China Plain (31). On the other hand, extreme weather events like droughts and floods induced by climate change may adversely impact crop production (30). In a warming environment, pests and crop diseases may also expand their geographic ranges, harming agricultural productivity. Coping with these uncertainties may require remediation strategies such as optimization of the entire bioenergy supply chains (32-34).

S2.2 Cost of crop residues

In the present analysis, we assume that each CBECCS system would require 10–

24 collection stations to process sufficient crop residues with an average collection radius of 10 km. The prices of crop residues (P_p) are determined primarily by collection costs (C_c), processing costs (C_P) and transportation costs (C_t), as follows.

S2.2.1 Collection cost

Following the methods of an earlier study by Zhang et al. (35), the total cost for collecting crop residues, C_c, can be calculated by:

$$C_c = [P_s + C_{w1} + (C_{d1} + C_{l1}) + C_{vd1} + C_{m1}] \cdot (1 + \theta) \quad (6)$$

$$C_{d1} = (q_{f1} + q_{e1}) \cdot d1 \cdot P_d \quad (7)$$

$$d1 = \frac{\int_0^R (2\pi r \cdot D \cdot 1.5r)dr}{\int_0^R (2\pi r \cdot D)dr} = \frac{\pi \cdot R^3}{\pi \cdot R^2} = R \quad (8)$$

With terms defined as follows. We assume the average distance of crop residues from their collection stations, R is 10 km, in which we further assume that the transport distance (*d1*) is 1.5 times the direct distance (*r*) to take account of zig-zags of roads between the residue supplies and collection stations; D refers to the density of crop residues; the price of purchasing straw, P_s=150.0 RMB/t; the cost of labor for shipping straw C_{w1}=40.0 RMB/t; the quantity of diesel consumed by a full-loaded vehicle, q_{f1}=0.063 L/(km·t), as summarized in Table S9) (36); the quantity of diesel consumed by an empty vehicle, q_{e1}=0.042 L/(km·t) (36); the price of diesel, P_d=6.5 RMB/L (see Table S10); the cost of lubricant consumed for shipping straw, C_{l1}=0.15C_{d1}; the cost of vehicle depreciation for transporting crop residues, C_{vd1}=2.2 RMB/t; the cost of vehicle maintenance, C_{m1}=0.5C_{vd1}; and the broker profit estimated as a percentage of all of the collection expenses above, θ =10%. The total cost of collecting residues is thus C_c= 221.3 RMB/t (35.6 US $/t).

S2.2.2 Cost of Processing and storage in collection station

The cost of processing and storage, C_P (RMB/t), is calculated by:

$$C_P = [C_{sw} + C_e + C_{sm} + h] \cdot (1 + \theta) \quad (9)$$

Here, we adopted the cost parameters from a study by Zhang et al. (35): the cost of labor at collection stations, C_{sw}=14.2 RMB/t; the cost of energy consumed in processing and storage, C_e= 32.5 RMB/t; the cost of collection station maintenance, C_{sm}=2.0 RMB/t; the cost of holding inventory, h= 17.8 RMB/t. This yields the cost of processing and storage, C_P=73.0 RMB/t.

S2.2.3 Transportation cost from collection station to CBECCS plants

The cost of delivery of crop residues from the collection stations to CBECCS plants is expressed as C_t (RMB/t):

$$C_t = [C_{w2} + (C_{d2} + C_{l2}) + C_{vd2} + C_{m2}] \cdot (1 + \theta) \quad (10)$$

$$C_{d2} = (q_{f2} + q_{e2}) \cdot d2 \cdot P_d \quad (11)$$

Based on the same study (35), we assume that the distance for transporting crop residues, *d2*=70 km; the cost of labor for shipping crop residues C_{w2}=8.3 RMB/t; the quantity of diesel consumed by a full-loaded vehicle, q_{f2}=0.0125 L/(km·t) (36); the quantity of diesel consumed by an empty vehicle, q_{e2}=0.0083 L/(km·t) (36); the price of diesel, P_d =6.5 RMB/L; the cost of lubricant consumed for shipping crop residues, C_{l2}=0.1C_{d2}; the cost of vehicle depreciation for shipping crop residues, C_{vd2} =1.8 RMB/t; the cost of vehicle maintenance for shipping crop residues, C_{m2}=0.5C_{vd2}; and the broker profit estimated as a percentage of all of the transportation expenses above, θ =10%. The cost of transporting crop residues is thus C_c= 23.6 RMB/t.

S2.2.4 Price of crop residues at CBECCS power plants

The price at the power plant, P_p, is:

$$P_p = C_c + C_P + C_t \qquad (12)$$

Based on the preceding estimation, P_p = 317.9 RMB/t (51.1 US$/t). In practice, the price of biomass in China available for use at power plants has been observed to be 310 RMB/t to 400 RMB/t, or 49.8 US $/t to 64.3 US $/t (35, 37). Compared with 70.0 US $/t in US, the lower prices of biomass in China are attributed primarily to lower labor costs (38), as collection and processing of crop residues consist of very labor-intensive activities.

S3 Life-Cycle GHG Emissions

S3.1 Goal and scope

The scope for the life-cycle analysis (LCA) includes all operations required for the production of biomass, coal mining and processing, coal and biomass transportation and maintenance, as well as the operation of the CBECCS system. Here we take wheat straw as a representative case for the LCA evaluation of GHG emissions from use of crop residues for energy in China. The production of auxiliary equipment contributes negligible emissions compared to those of other included sources and are not taken into account the present analysis (39). The reference unit selected here is one kWh of generated electricity delivered to the grid. In addition, the R/G index on an energy basis is set as 1.2 following current literature (40).

S3.2 Upstream GHG emission of crop-residues

S3.2.1 Direct and indirect GHG emissions of crop (wheat) production

The major direct GHG emissions associated with wheat production involve CO_2

emissions associated with the loss of soil organic carbon (SOC) and N_2O emissions resulting from application of nitrogen fertilizer. Using an SOC of 377.2 kg/(ha·yr), the average value for the three largest wheat-producing provinces (Hebei, Henan and Shangdong) in China (26, 41, 42), we estimate the direct CO_2 emission factor for wheat production as 0.31 t CO_2-eq /t wheat. According to IPCC 2006 Guidelines, the N_2O emissions can be evaluated by using a combination of factors including nitrogen fertilizer usage for wheat cultivation (608.6 kg N fertilizer /(ha·yr)) (43), the nitrogen content of nitrogen fertilizer of 300 g/kg, the IPCC default value of conversion factor from nitrogen content to N_2O emissions (0.01 kg N_2O/kg N) in dry land, together with the wheat production per hectare crop field (43-45). The N_2O emissions associated with wheat planting are estimated at 0.39 g/kg wheat, equivalent to 0.12 t CO_2-eq /t wheat. The direct GHG emissions associated with crop production are summarized in Table S11.

Indirect emissions refer to emissions resulting from the production of materials and energy required for agricultural activities, which include fertilizers, pesticides, equipment and diesel, as summarized in Table S11 (46-48). This study mainly considers three major GHGs associated with crop-residue biomass, namely CO_2, CH_4 and N_2O. For global warming potential on a timescale of 100 years, 1 g of CH_4 and 1 g of N_2O are equivalent to 25 g and 298 g of CO_2, respectively (48, 49).

S3.2.2 GHG emissions during collection and transportation of crop residues

Crop residues on farm land would be collected and transported to collection stations, and after initial processing, they would be finally delivered by trucks to

CBECCS power plants. The diesel consumption rate of trucks is listed in Table S9, and the associated GHG emissions during collection and transportation of biomass are summarized in Table S10. The present analysis differentiates the GHG emissions from the loaded and unloaded modes of trucks during their round trips.

In addition, biomass is lost (wasted) during collection, storage and transportation and this would incur an increase of pre-combustion emissions per unit delivered biomass. The overall loss rates reported in literature vary from 3% to 10.9% during transportation and storage, relative to the initial dry matter (50, 51). In the present analysis, we adopted a loss rate of 5% of the crop residue under transport and storage conditions in the LCA of GHG emissions. As calculated in Table S11, the overall pre-combustion GHG emissions of crop residue (wheat) would increase to 15.6 g CO_2-eq/MJ including consideration of the losses.

S3.3 Upstream GHG emissions of coal

The upstream GHG emissions for coal delivered to power plants include not only the CO_2 emissions associated with the energy consumed in the mining, extraction and transportation processes, but also the methane released during mining and extraction (52). Data for coal mine commissioning and construction are from the Mining Engineering Design Handbook (53). Data for coal mining operations are collected from the China Coal Industry Yearbook (54). The average electricity consumption for mining processes is 26.7 kWh/t coal, and coal consumption is 10.9 kg/t coal (39). Data for coal transportation are based on the China Traffic Yearbook (39, 55). In 2005, diesel locomotives were used for 57.3% of total coal transportation, for which the diesel use

was 24.6 kg/(10^4t·km). The remaining 42.7% of coal is transported by electric locomotives, which consumes 111.8 kWh/(10^4t·km) (39). As calculated in Table S12, a value of 9.5 g CO_2-eq/MJ is adopted for pre-combustion GHG emissions for coal in the present analysis, compared to a the range of 7.3 g CO_2-eq/MJ to 29.0 g CO_2-eq/MJ reported in existing literature (52, 56).

S3.4 Combustion GHG emissions

The combustion of crop residues in CBECCS systems is deemed not to contribute additional CO_2 emissions, as their organic carbon was derived from CO_2 in the atmosphere through photosynthesis in the growing process.

Carbon emissions from coal combustion are calculated per kWh of electricity generation. The data of net generating efficiency, emissions before flue-gas cleaning systems, and the amounts of flue gas for a full load of coal fired power plants are derived from operational reports and feasibility studies of currently operating power plants (39). As summarized in Table S13, the operational data for IGCC and CBECCS systems used in this study are derived from the Tianjin IGCC power plant (39) . The CO_2 emissions per kWh electricity generated by the CBECCS systems vary also as a function of biomass ratios and the associated net efficiency for electricity generation, and are estimated through the Aspen Plus simulation.

S3.5 GHG emissions associated with plant construction

The power plant construction data are collected from the Thermal Power Engineering Design Handbook (39, 57). The IGCC construction data are collected from U.S. National Energy Technology Laboratory reports (39, 58). Here, we assume that

the carbon emissions associated with CCS facility construction are the same as those of the IGCC system on the basis of per capital investment. With assumptions of plant lifetimes of 35 years and capacity factors of 80%, the CO_2 emissions associated with plant construction are 0.55 g CO_2-eq/kWh, 0.81 g CO_2-eq/kWh, 0.88 g CO_2-eq/kWh respectively for IGGC, supercritical pulverized coal plants (SC-PC), and CBECCS systems (see Table S13).

S4 Economic analysis

The levelized cost of electricity generation (LCOE) using the CBECCS systems is evaluated using a cash flow model with considerations of overnight capital investment, operational and maintenance (O&M) costs, and expense for fuel (14). The overnight capital cost includes both total plant cost (TPC) and owner's cost (OC). The TPC here refers to expense for site preparation, and the costs of the equipment for fuel preparation, ASU, gasification, WGS, AGR, gas and steam turbines and their related devices. Given little literature discussing the ratio of the OC/TPC ratio in China, we opted to follow results reported by the US National Energy Technology Laboratory (NETL) and took 13.6% for the OC/TPC ratio in the present analysis (15). Following the financial parameters reported by the Asian Development Bank (ADB), we adopted the overnight capital cost of 2085.0 $/kW for the CBECCS-Br0 with a CO_2 capture rate of 90%, and the capital costs of 234.1 $/kW and 214.0 $/kW respectively for CO_2 transport and storage (5). The construction period is assumed to be three years, with the first, second and third years allocated respectively with 20%, 60% and 20% of the total capital investments (see Table S14).

The O&M cost includes expenses associated with operating and maintaining the power plants over their expected lifetimes, which can be classified into fixed and variable O&M costs. Based on the ADB and NETL for IGCC-CCS system, the fixed and variable O&M costs are chosen as 113.5 \$/kW/yr and 9.1 \$/MWh respectively (Table S14). The fixed and variable O&M costs of CO_2 transport and storage are also shown in Table S14. The higher the biomass ratio, the larger the volume of feedstock need to be handled, and therefore the larger the size of gasifier and the more labor that is required (59, 60). In particular, the capital investment and fixed O&M costs are assumed to increase respectively by 1% and 3% for every 10% increment of biomass ratio, due to the larger volume of feedstock (61, 62). In the case of biomass blends, more labor is required for the feedstock treatment and more oxygen is needed to reach a suitable temperature inside the gasifier. The price for coal is selected as US \$ 80 /ton, reflecting the average coal prices in 2017 and 2018 for typical power plants in the Bohai region of China (63). The costs of biomass are discussed and estimated in section S2. The cash flow model (64) used to evaluate the LCOE for CBECCS systems is expressed through equations (13) and (14):

$$C_{total} = C_{cap} \cdot \left[\frac{r(1+r)^n}{(1+r)^n-1}\right] \cdot (1+r)^t + \mathrm{F}_{OM} + V_{OM} + C_{fuel} + C_{tax} \quad (13)$$

$$\mathrm{LCOE} = \frac{C_{total}}{E} \quad (14)$$

where C_{total} is the total annual cost; C_{cap} (US\$) is the capital cost of the plant; r is the discount rate; n (yr) is the operational lifetime; t (yr) is the construction time; F_{OM} (US \$/kW/yr) and V_{OM} (US\$/MWh) indicate the annual fixed and variable O&M costs, respectively; C_{fuel} (US \$) is the annual fuel cost; C_{tax} (US \$) is the

annual carbon tax expenditure; and E (MWh) is the annual electricity generation. The value of r in this study is chosen as 7% to reflect the social return rate (65). The breakdown of LCOE for these cost elements is shown in Fig. S2 for coal-fired power plants and CBECCS systems with and without carbon taxes. Sensitivity analyses of the LCOE in response to variations in capital costs, fixed O&M, variable O&M social discount rate and fuel prices are illustrated in Fig. S3.

S5 Potential co-benefits to air pollution

The benefits of CBECCS system in reducing the emissions of air pollutants come from two aspects: one is rooted in the reduction of open field (OBB) and domestic (DBB) biomass burning, and the second is associated with the emissions mitigated through the CBECCS process.

S5.1 Biomass burning

Open and domestic biomass burning (OBB and DBB) are still common ways to dispose of crop residues after harvests in China. Previous studies estimated that 17-25.6% and 40% of China's crop wastes were burned directly in open field and in households, respectively, resulting in emissions of a number of air pollutants such as SO_2, NO_x, particulate matter (PM), and volatile organic compounds (VOCs) (66, 67). The emissions from open field burning of crop residues can be estimated on the basis of crop yield, R/G index, burning efficiency and emission factors as follows:

$$\mathrm{M}_c = M_p \cdot \mathrm{C}_p \cdot \mathrm{C}_b \cdot \mathrm{C}_e \quad (15)$$

where M_c refers to the air pollutants emitted from the direct burning of crop residues; M_p is the total crop yield in China (as discussed in section S2.1 on biomass

distribution); C_p is the R/G index for crops residues (Table S7); C_b (%) is the direct burning ratio for crop residues; and C_e (g/kg) is the emission factor for corresponding air pollutants produced in burning of crop residues. The open burning ratio (C_b) for crop residues is taken as 20% (68). Emission factors for biomass open burning, C_e, are derived from previous studies (66, 69, 70) and summarized in Table S15. When the crop residues are used in CBECCS systems, air pollutant emissions from OBB discussed above would be avoided.

In addition, the demand for crop residues of CBECCS system is expected to reduce the burning for household use (i.e., DBB), contributing to an additional reduction in air pollutants should crop residues be replaced by clean energy. Here, the burning ratio of DBB is chosen as 40%.

S5.2 CBECCS system

In contrast to traditional pulverized coal-fired (PC) power plants, nearly all of the particulates, mercury, and compounds containing nitrogen or sulfur can be removed from syngas before combustion in CBECCS systems, offering an effective way to reduce emissions of air pollutants (14). As a result, per kWh emissions of SO_2, NOx, $PM_{2.5}$ and black carbon (BC) from a CBECCS plant are significantly lower compared to those of traditional PC plants (Table S16). The emission reduction in air pollutant *k* by CBECCS system (M_{CBECCS}^{k}) can be calculated as follows:

$$M_{CBECCS}^{k} = \left(C_{PC}^{k} - C_{CBECCS}^{k}\right) \cdot E_{CBECCS} \tag{16}$$

where C_{PC}^{k} (g/kWh) and C_{CBECCS}^{k} (g/kWh) are the emission factors of air pollutant *k* associated with production of one kWh of electricity respectively by PC and

CBECCS power plants; and E_{CBECCS} (kWh) represents electricity generation by CBECCS displacing that of PC plants. In particular, k represents air pollution species of SO_2, NO_x, primary $PM_{2.5}$ and BC.

S6 Spatial distribution of crop residues, air pollution and carbon storage capacity in China

The spatial patterns of crop residue availabilities and air pollutant emissions provide a preliminary guideline for the deployment of the CBECCS systems discussed in this analysis. The amount of crop residues by province is calculated based on crop yields from the NBSC and the R/G index for each type of crops derived from existing studies (23, 25). As illustrated in Table S17, crop residues especially concentrated in major grain producing areas one of which includes Henan, Shandong and Hebei provinces amounting respectively to 100.89 Mt, 84.39 Mt and 62.67 Mt annually. The annual yields of rice and wheat straw crop residues in Anhui and Jiangsu are also relatively high, amounting respectively to 44.17 Mt and 38.18 Mt. The northeast provinces of Liaoning, Jilin and Heilongjiang, as a main production area for corn and rice, respectively yield 36.61 Mt, 66.72 Mt and 86.87 Mt of crop residues annually. The potential storage capacity for CO_2 is mainly distributed among seven regions of China (see Table S18), namely Huabei, Dongbei, Yuwan, Ordos, Jianghan-Dongting, Sichuan and Xinjiang basins (see Table S18). Huabei and Yuwan basins, covering the same Hebei, Henan, Shandong and Anhui provinces that produce a lot of crop residues, have abundant sequestration capacities of CO_2, which are estimated at 264 Gt and 186 Gt. Given these capacities, the deployment of 116 CBECCS power plants in the proposed

provinces theoretically would not face limits of carbon storage capacity for more than 2000 years.

The highest pollution emissions occur in the eastern part of China, particularly in the North China Plain and the Yangtze River Delta (71). As shown in Table S17, Shandong, Henan, Hebei and Anhui are among the top ten provinces for emissions of SO_2, NO_x, $PM_{2.5}$ and black carbon. Previous studies have demonstrated that burning of fossil fuel and biomass are the most important sources for air pollution in these regions (71-73). As shown in Fig. S4, large amounts of coal-fired power generation are found also in these four provinces, where locates the top ten provinces in terms of annual crop residue yield. Shandong, Henan, Hebei and Anhui thus appear to offer particularly strong potential for air pollution abatement with the deployment of CBECCS systems.

Appendix 1: Tables

Table S1: Design parameters in Aspen Plus simulation for CBECCS systems.

Unit	Parameters
Air separation unit (ASU)	Oxygen purity: 95% (vol.)
	Oxygen and Nitrogen delivery pressure: 2.37 bar
	ASU power consumption: 225 kWh/ton O_2
	Additional gas compression efficiency: 85%
Coal and biomass preparation and handling	Electric power consumption of coal: 0.29% of input coal
	Biomass handling: 20 kJ/kg biomass
	Aspen plus module: Crusher
Gasification plant (Entrained-flow)	Steam/coal ratio (kg/kg): 0.12
	Steam/biomass ratio (kg/kg): 0.068
	O_2 pressure to gasifier: 40 bar
	Gasification pressure: 40 bar
	Gasification temperature: 1400 ^{o}C by adjusting O_2 input
	Carbon conversion: 99%
	Pressure drop: 1.5 bar
	Electric power consumption: 1% of input fuel LHV
	The total amount of feedstock energy: ~1112 MW by adjusting biomass input
	Aspen plus module: RYIELD, RGIBS, Separation column

Syngas quench	Cooling type: water scrubber<br>Temperature after quench: ~ 210 ºC
Water gas shift (WGS)	Sulfur tolerant catalyst (sour shift)<br>Two shift stages (fixed-bed)<br>First stage thermal mode: adiabatic<br>Second stage thermal mode: Isothermal<br>No external steam input required in either stage<br>Pressure drop: 3 bar<br>CO conversion: ~ 98%<br>Aspen plus module: RStoic
Acid gas removal (AGR) and CO_2 compression	Solvent: Rectisol® (low temperature methanol washing)<br>Separate H_2S and CO_2 capture<br>H_2S removal rate: 99.9%<br>CO_2 removal rate: ~ 80%<br>H_2S recovery: Thermal processed and sent to Claus<br>CO_2 recovery: Pressure drop and compression (150 bar)<br>Aspen plus module: Absorber, distillation, RStoic
Gas turbine	Turbine type: M701G2<br>Isentropic efficiency: 90%<br>Gas input temperature: 1400 ºC

	Gas outlet temperature: ~ 610 ºC
	Aspen plus module: RGIBS, Compressor, Heater
Heat recovery steam generator (HRSG)	Three pressure levels: 118 bar (HP); 34 bar (MP); 3 bar (LP)
	LP turbine exhaust pressure: 0.046 bar
	Steam turbine isentropic efficiency: 85%
	Aspen plus module: Heat exchanger, Compressor
Heat exchangers	Pressure drop: 1% of input pressure
	$\triangle T_{min}$ = 10 ºC (gas-liq), 20 ºC (gas-gas)

Table S2: Key performance indicators for the five CBECCS systems.

System name	CBECCS-Cr	CBECCS-CrB1	CBECCS-CrB2	CBECCS-CrB3	CBECCS-CrB4
Biomass mass ratio %	0	20	35	70[a]	100
Gross electricity efficiency (%)	48.88	49.09	49.16	49.44	46.82
Net electricity efficiencies (%)	37.06	37.28	37.35	37.67	34.92
Carbon capture rate (%)	87.71	87.70	87.69	87.65	87.58
Life-cycle GHG emission (kg/MWh)	214.94	105.40	14.73	-261.45	-650.94
Comments	Pure coal yields high CO_2 emissions	20% biomass ratio yields zero CO_2 emissions	35% biomass ratio yields almost zero life-cycle CO_2-eq emissions	70% biomass ratio yields negative CO_2 emissions	Pure biomass yields negative CO_2 emissions

[a] This biomass ratio considers the size of the availability of feedstock and road congestion issue in feedstock supply (74).

Table S3: Main characteristic of plant streams for CBECCS-CrB0.

Steam	Coal	Biomass	Oxygen (gasification)	Water (gasification)	Raw syngas	Quenched syngas	Shifted syngas	H_2-rich gas	Nitrogen (to GT)	Flue gas (ex.GT)
Temperature (ºC)	Ambient	Ambient	411.59	15.14	1399.96	205.48	305.01	30.00	339.78	612.21
Pressure (bar)	Ambient	Ambient	40.00	40.00	38.50	37.50	31.50	28.00	25.50	1.00
Molar flow (kmol/h)			3231.54	1092.24	14027.02	25895.41	29751.65	13255.76	7100.00	88009.41
Mass flow (kg/h)	163975		104222	19677	279218	493026	562497	79721	199147	2404550
Composition (% vol.):										
N_2			1.96		0.73	0.40	0.35	0.73	99.59	75.39
H_2					31.21	16.91	40.06	89.69	0	0
O_2			93.88		0	0	0	0	0.17	9.02
H_2O				100	7.90	50.11	31.24	0	0	13.51
AR			4.16		0.96	0.52	0.45	0.91	0.24	0.76
CO					54.85	29.71	0.52	1.07	0	0
CO_2					4.25	2.30	27.35	7.54	0	1.30
SO_2					0.01	0	0	0	0	0
COS					0.02	0.01	0.01	0	0	0
H_2S					0.07	0.04	0.04	0	0	0

Table S4: Main characteristic of plant streams for CBECCS-CrB2

Steam	Coal	Biomass	Oxygen (gasification)	Water (gasification)	Raw syngas	Quenched syngas	Shifted syngas	H_2-rich gas	Nitrogen (to GT)	Flue gas (ex. GT)
Temperature (ºC)	Ambient	Ambient	411.59	15.11	1400.84	214.03	304.98	30.00	339.78	608.71
Pressure (bar)	Ambient	Ambient	40.00	40.00	38.50	37.50	31.50	28.00	25.50	1.00
Molar flow (kmol/h)			3373.03	1055.79	15416.75	29232.39	32578.44	12938.43	7100.00	86250.40
Mass flow (kg/h)	118000	78245	108749	19020	308716	557609	617889	78730	199147	2357390
Composition (% vol.):										
N_2			1.88		0.68	0.36	0.32	0.75	99.59	75.44
H_2					29.78	15.71	35.64	89.53	0	0
O_2			94.14		0	0	0	0	0.17	9.04
H_2O					14.97	55.16	38.22	0	0	13.43
AR			3.99		0.87	0.46	0.41	0.91	0.24	0.76
CO					46.45	24.50	0.44	1.01	0	0
CO_2					7.14	3.77	24.92	7.73	0.00	1.31
SO2					0.01	0	0	0	0	0
COS					0.01	0	0	0	0	0
H_2S					0.09	0.05	0.04	0	0	0

Table S5: Key performance indicators for CBECCS CrB0-CrB4

Main Plant Data	Units	CBECCS-CrB0	CBECCS-CrB1	CBECCS-CrB2	CBECCS-CrB3	CBECCS-CrB4
Coal flow	kg/h	163975	142500	124500	69000	0.1
Crop residue flow	kg/h	0	36548.63	67182.88	161639.00	279070
Coal energy rate	%	100.00	86.90	75.93	42.08	0
Crop residue energy rate	%	0	13.10	24.07	57.92	100.00
Coal mass rate	%	100.00	79.59	64.95	29.92	0
Crop residue mass rate	%	0	20.41	35.05	70.08	100
Feedstock thermal energy LHV (A)	MW	1100	1100	1100	1100	1100
Thermal energy of syngas (B)	MW	899.84	886.74	877.21	856.48	827.48
Cold gas efficiency (B/A*100)	%	81.80	80.61	79.75	77.86	75.23
Thermal energy of syngas exit AGR (C)	MW	814.44	804.21	796.92	780.79	757.04
Syngas treatment efficiency (C/B*100)	%	90.51	90.69	90.85	91.16	91.49
Gas turbine output	MW	373.13	368.69	365.56	358.81	348.78
Steam turbine output	MW	164.58	171.29	175.19	185.03	166.29
Gross electric power output (D)	MW	537.72	539.98	540.75	543.84	515.07
ASU consumption and O_2 compression	MW	34.36	35.08	35.57	36.57	38.06
Gasification island power consumption	MW	14.05	13.92	13.80	13.43	12.87
AGR and CO_2 compression	MW	62.47	61.77	61.32	60.31	60.89
Power island power consumption	MW	19.19	19.18	19.17	19.16	19.16
Total ancillary power consumption (E)	MW	130.07	129.94	129.86	129.47	130.97
Net electric power output (F=D-E)	MW	407.65	410.04	410.89	414.37	384.09

Gross electrical efficiency (D/A*100)	%	48.88	49.09	49.16	49.44	46.82
Net electric LHV efficiency (F/A*100)	%	37.06	37.28	37.35	37.67	34.92
Net electric HHV efficiency	%	35.70	35.60	35.43	35.18	32.16
Carbon capture rate	%	87.71	87.70	87.69	87.65	87.58
CO_2 specific emissions	kg/MWh	122.75	6.53	-89.98	-383.75	-807.66

Table S6: Feedstock characteristics for coal and biomass (24).

	Inner Mongolia bituminous	Wheat straw
Proximate Analysis (% wt. as-received)		
Fixed Carbon	53.19	13.92
Volatile Matter	28.29	59.50
Ash	4.24	11.58
Moisture Content	14.29	15
Lower Heating Value (MJ/kg)	24.15	14.19
Ultimate Analysis (% wt. dry basis)		
Carbon	70.65	40.39
Hydrogen	4.69	6.26
Oxygen	19.38	38.85
Nitrogen	0.79	0.56
Chlorine	0	0
Sulfur	0.25	0.31
Ash	4.94	13.63
Calorific value (MJ/kg, dry basis)		
Lower Heating Value (MJ/kg)	28.18	16.69
Higher Heating Value	29.25	18.12

Table S7: The Residue to Grain index and LHV for crops residues (23-25).

Agriculture groups	Rice	Wheat	Corn	Beans	Tubers	Cotton	Oil crops	Sugarcane	Other grains
Residue to Grain index (R/G index, dry-basis)	0.62	1.2	2	1.5	1	3	2	0.1	2.5
LHV (MJ/kg, dry-basis)	14.61	16.69	16.99	17.19	16.72	17.89	19.06	16.65	16.26

Table S8: The crop production, crop residue resource, electric generation capacity, number of plants, and crop residue collection area in China for the CBECCS-CrB2 system.

Region	Crop (Mt, dry-basis)	Available crop residues (Mt, dry-basis)	Crop residues requirement (Mt, dry-basis)	CBECCS-CrB2 capacity (GW)	Number of Plants	Capacity factor (%)	Crop residue collection area (100 km^2)
North China	86.01	148.44	36.08	24.32	59	80	16.98
Northeast	120.84	190.20	46.23	30.71	75	80	14.41
East China	163.66	196.81	47.84	31.43	77	80	24.27
South Central China	252.12	197.91	48.11	31.73	77	80	26.93
Southwest	103.15	108.64	26.41	17.45	43	80	29.98
Northwest	55.48	87.26	21.21	14.36	35	80	22.33
Total	781.26	929.26	225.88	150	366	80	

Table S9 Diesel consumption rate by trucks in China (36).

	Unit	0-2t	2-4t	4-8t	8-20t	20t +
Average load capacity	t	1.2	3.2	5.7	14	28
Load capacity utilization rate		90%	110%	120%	130%	140%
Fuel consumption (loaded)	L/100 km	15.1	20.2	25.1	30.7	35
Fuel consumption (unloaded)	L/100 km	10.1	13.5	16.7	20.5	23.3

Table S10 Energy, GHGs and price of diesel consumed directly in agricultural transport (75).

Fuel type	Energy (MJ/L)	CO_2 (kg/L)	CH_4 (g/L)	N_2O (g/L)	GHG intensity (kg CO_2-eq/L)	Cost (RMB/L)	Cost (US$/L)
Diesel	35	2.7	0.14	0.14	2.78	6.5	1.05

Table S11 GHG emissions in agricultural processes and transportation.

Agricultural processes	GHG intensity ((t CO_2-eq/t))	Consumption per wheat ((t/t wheat))	GHG emission ((t CO_2-eq/t wheat [a]))
Indirect			
Nitrogenous fertilizer (40, 49)	1.64	2.97E-02	4.87E-02
Phosphatic fertilizer (40, 49)	1.05	1.40E-02	1.47E-02
Potash fertilizer (40, 49)	0.03	3.09E-03	9.26E-05
Pesticide (40, 49)	3.00	4.20E-04	1.26E-03
Equipment (76)	1.39	1.16E-03	1.61E-03
Diesel (76)	0.45	2.37E-03	1.07E-03
Direct (26, 41-45)			
CO_2			3.08E-01
CH_4			0.00E+00
N_2O			1.17E-01
Subtotal			4.87E-01
Subtotal with allocation based on energy			2.41E-01
Collection and transportation processes (75)	GHG intensity (t CO_2-eq/ (t·km))	Distance (km)	GHG emission (t CO_2-eq/t wheat straw)
Collection			
Diesel	2.9E-04	10	2.93E-03
Transportation			
Diesel	5.8E-05	70	4.09E-03
Subtotal			7.02E-03
Total processes			
Total emission (t CO_2-eq/t)			2.48E-01

Total emission (g CO_2-eq/MJ)	1.48E+01
Total emission (g CO_2-eq/MJ)[b]	1.56E+01

a. The quantity of wheat straw based on dry-basis.

b. The total emission considering the loss during transportation and storage.

Table S12 Upstream GHG emissions of crop residue as compared with coal (39).

Biomass	Emission intensity ($g\ CO_2$-eq/MJ)	Coal	Emission intensity ($g\ CO_2$-eq/MJ)
Indirect agricultural processes	2.00	Mine commissioning	0.00
Direct agricultural processes	12.38	Mine construction	0.03
Collection processes	0.18	Mine operation	7.85
Transportation processes	0.26	Transport construction	0.04
Biomass loss	0.78	Transportation of coal	1.56
Total	15.60	Total	9.49

Table S13 GHG emission during construction and operation for IGCC, SC-PC and IGCC-CCS (39).

Process (g CO_2-eq/kWh)	IGCC	SC-PC	IGCC-CCS
Plant construction	0.55	0.81	0.88
Plant operation	723	774	122
Capacity factor (%)	80	80	80
Total	723.55	774.81	122.88

Table S14 Economic parameters different power generation systems.

	SC-PC (5)	IGCC (5)	CBECCS-CrB0 (5, 15)	CBECCS-CrB2 (5, 15)	IGCC from NETL (15)	IGCC-CCS from NETL(15)
Total plant cost ($/kW)	690	1395	2229	2306	2578	3860
Owner's cost ($/kW)	94	190	304	315	351	526
Overnight Capital cost ($/kW)[a]	784	1585	2533	2621	2929	4386
Fixed O&M ($/kW/yr)	31.35	68.32	113.53	125.45	94.78	133.96
Capacity factor (%)	80	80	80	80	80	80
Variable O&M ($/MWh)[b]	3.62	3.69	9.09	9.09	9.23	22.73
Coal cost ($/MJ, LHV basis)	3.31	3.31	3.31	3.31	2.90	2.90
Biomass cost ($/MJ, LHV basis)	3.52	3.52	3.52	3.52	4.93	4.93
Construction time/Lifetime (yr)[c]	3/35	3/35	3/35	3/35	3/35	3/35
Net power LHV efficiency (%)	42.56	45.56	37.06	37.35	41.20	32.20

a The overnight capital cost includes both total plant cost (TPC) and owner's cost (OC).

b The variable O&M for SC-PC, IGCC, CBECCS-CrB0 and CrB2 in China are assumed as 60% of the NETL data (77).

c The construction period is assumed to be three years and 20%, 60% and 20% of the capital costs are allocated respectively for the first, second and third years.

Table S15 Emission factors for open biomass burning (OBB) and domestic biomass burning (DBB) (69).

Sample (kg/t)	SO_2	NO_x	PM_{10}	$PM_{2.5}$	BC[a]
Domestic burning (DBB)					
Rice	0.53	0.42	3.32	1.66	0.066
Wheat	0.53	0.86	11.22	5.61	0.22
Corn	0.53	0.76	4.90	2.45	0.098
Cotton	0.53	1.29	12.08	6.04	0.24
Sugarcane	0.53	0.90	12.54	6.27	0.25
Other	0.53	1.29	7.88	3.94	0.16
Opening Burning (OBB)					
Rice	0.53	1.29	19.3	9.65	0.37
Wheat	0.85	3.30	15.2	7.60	0.30
Corn	0.44	4.30	23.4	11.7	0.47
Cotton	0.53	1.29	19.3	9.65	0.39
Sugarcane	0.53	1.29	19.3	9.65	0.39
Other	0.53	1.29	19.3	9.65	0.39

[a] The emission factor of BC is evaluated based on the emission factor of $PM_{2.5}$.

Table S16 Emission factors of air pollutants for different power generation technology (78).

	Sub-critical PC[a] (g/kWh)	Supercritical PC[a] (g/kWh)	Ultra-supercritical PC[a] (g/kWh)	IGCC[a] (g/kWh)	CBECCS-CrB2[b] (g/kWh)
SO_2	0.982	0.934	0.870	0.054	0.008
NO_x	0.771	0.733	0.683	0.228	0.180
PM	0.298	0.283	0.264	0.049	0.026
Net power LHV efficiency (%)	40.48	42.56	45.67	45.56	37.35

a. The pollutant emissions for PC and IGCC are calculated from the average emissions in China in 2015

b. The pollutant emissions for CBECCS-CrB2 are derived from the NETL

Table 17 Top ten provinces for electricity demand, thermal power generation, coal consumption, available biomass, and air pollutant emissions.

Ranking	Electricity demand	Thermal generation	Coal consumption	Available crop residue	SO_2 emissions	NO_x emissions	$PM_{2.5}$ emissions	BC emissions
1	Guangdong	Jiangsu	Shandong	Henan	Shandong	Shandong	Shandong	Shandong
2	Jiangsu	Shandong	Shanxi	Shandong	Guizhou	Guangdong	Hebei	Hebei
3	Shandong	Inner Mong.	Inner Mong.	Heilongjiang	Henan	Henan	Henan	Henan
4	Zhejiang	Guangdong	Hebei	Jilin	Shanxi	Hebei	Jiangsu	Jiangsu
5	Hebei	Henan	Jiangsu	Hebei	Inner Mong.	Jiangsu	Anhui	Anhui
6	Henan	Shanxi	Henan	Inner Mong.	Sichuan	Anhui	Hunan	Hunan
7	Inner Mong.	Zhejiang	Shanxi	Anhui	Hebei	Inner Mong.	Heilongjiang	Heilongjiang
8	Liaoning	Hebei	Liaoning	Sichuan	Hubei	Zhejiang	Inner Mong.	Inner Mong.
9	Sichuan	Anhui	Guangdong	Xinjiang	Hunan	Sichuan	Shanxi	Shanxi
10	Xinjiang	Xinjiang	Xinjiang	Jiangsu	Jiangsu	Liaoning	Guangxi	Guangxi

Table S18 Regional CO_2 storage capacity, biomass supply and the number of CBECCS-CrB2 power plants envisaged in this study, the electricity supply, and pollutant emissions.

Region	Total CO_2 storage capacity (Gt) [a]	Oil field CO_2 storage capacity (Gt)	Biomass supply (PJ)	Number of CBECCS-CrB2 power plant [b]	Biomass required for CBECCS-CrB2 (PJ)	CO_2 storage capacity required for CBECCS-CrB2 (Mt)	Electricity demand (TWh)	Percentage of thermal power (%)	SO_2 emissions (kt)	NO_x emissions (kt)	$PM_{2.5}$ emissions (kt)
Huabei basin [c]	264.18	1.90	3611.17	175	880.77	186.42	1431.81	80.1	4393.06	5399.30	2315.75
Dongbei basin [d]	360.19	1.60	1323.57	124	322.82	68.33	156.43	123.0	593.44	1040.72	827.84
Yuwan basin [e]	186.07	0.07	2569.91	119	626.81	132.67	478.41	86.0	1584.78	2343.78	1175.00
Ordos basin [f]	258.47	0.36	2450.97	64	597.80	126.53	396.21	92.8	1550.96	1416.83	650.47
Jianghan-Dongting basin [g]	52.82	0.02	950.73	46	231.89	49.08	325.88	51.0	1733.81	1445.97	858.41
Sichuan basin [h]	78.67	0.02	1009.05	49	246.11	52.09	302.59	29.2	1620.99	1225.81	541.27
Xinjiang basin [i]	998.15	0.39	1286.55	31	313.79	33.21	231.65	88.9	418.34	623.94	269.28

a. data source from Li et al. b with assumption of CO_2 capture rate at 90%; c. covering Hebei, Beijing, Tianjin, Sanxi, Liaoning, and Shandong provinces; d. covering Jilin and Heilongjiang provinces; e. covering Henan and Anhui provinces; f. covering Inner Mongolia and Shanxi provinces; g. covering Hunan and Hubei provinces; h. covering Chongqing and Sichuan province; and i. covering Xinjiang.

Appendix 2: Figures

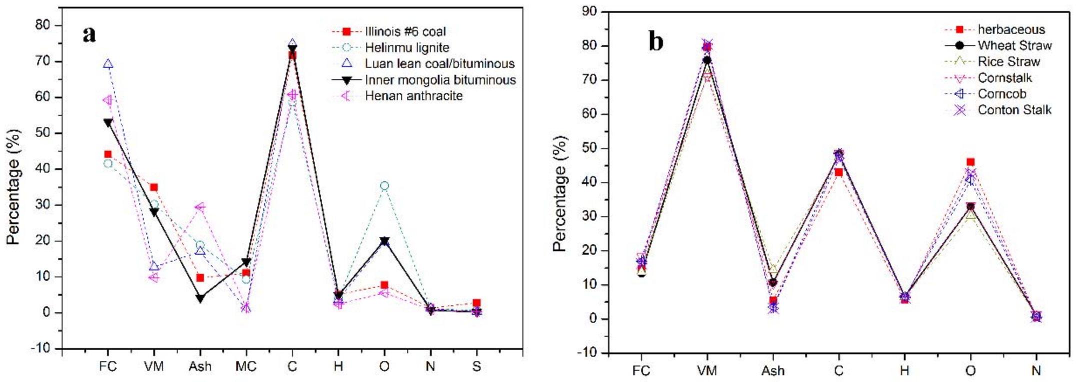

Fig. S1 The composition of various coal and crop fuels in China with respect to the proximate and ultimate analysis. (a): coal; (b): biomass. (FC: Fixed carbon; VM: volatile matter; MC: Moisture content)

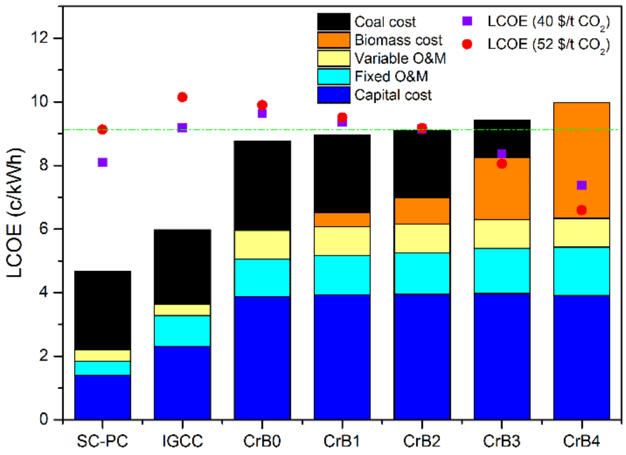

Fig. S2 Levelized cost of electricity (LCOE) of building and operating SC-PC, IGCC, and CBECCS power plants without a carbon tax (bars) and with a carbon tax at \$40/t CO_2 (purple squares) and \$52.0/t CO_2 (red circles). The costs for plants without a carbon tax are broken down for capital cost, the fixed and variable O&M costs, and coal and crop residue costs.

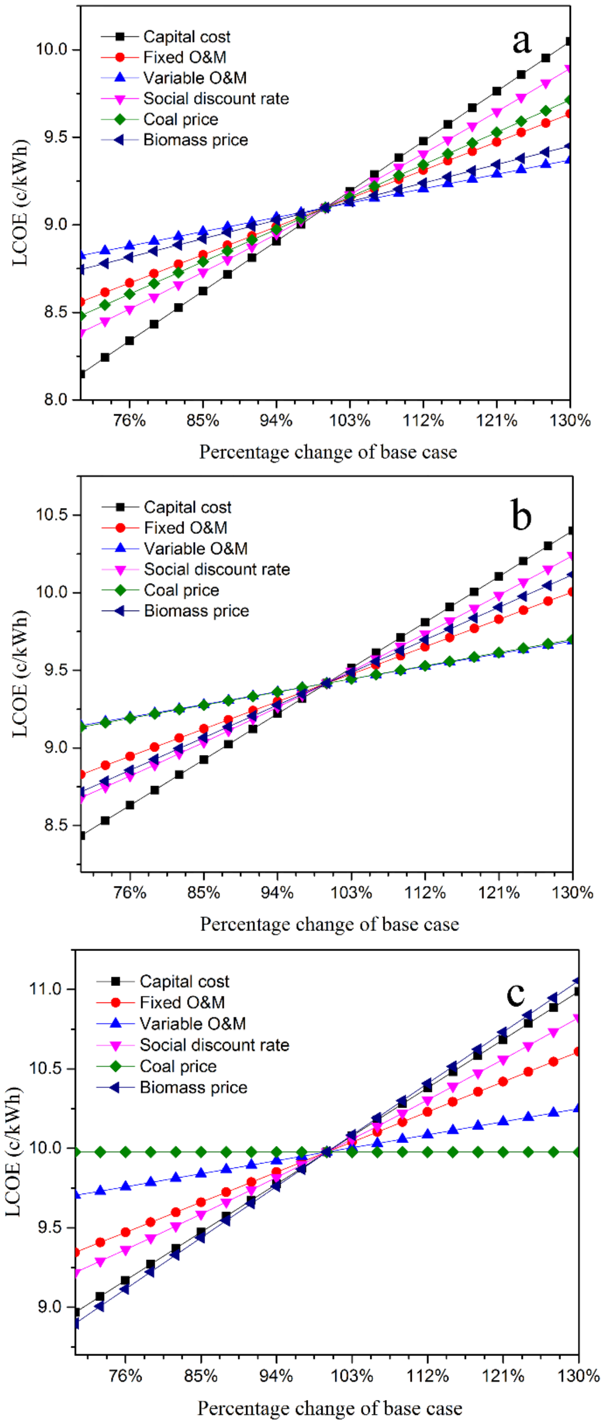

Fig. S3 Sensitivity analyses of various input parameters including capital cost, fixed O&M cost, variable O&M cost, social discount rate, coal price and biomass price, for (a): CBECCS-CrB2; (b): CBECCS-CrB3; (c): CBECCS-CrB4.

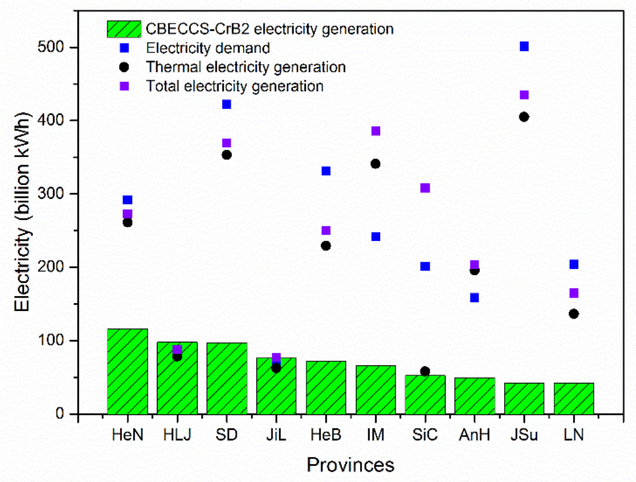

Fig. S4 Electricity generation and demand of the top ten provinces in terms of total annual yield of crop residues. (HeN: Henan; SD: Shandong; HLJ: Heilongjiang; HeB: Hebei; JiL: Jiling; AnH:Anhui; IM: Inner Mongolia; SiC: Sichuan; LN: Liaoning)

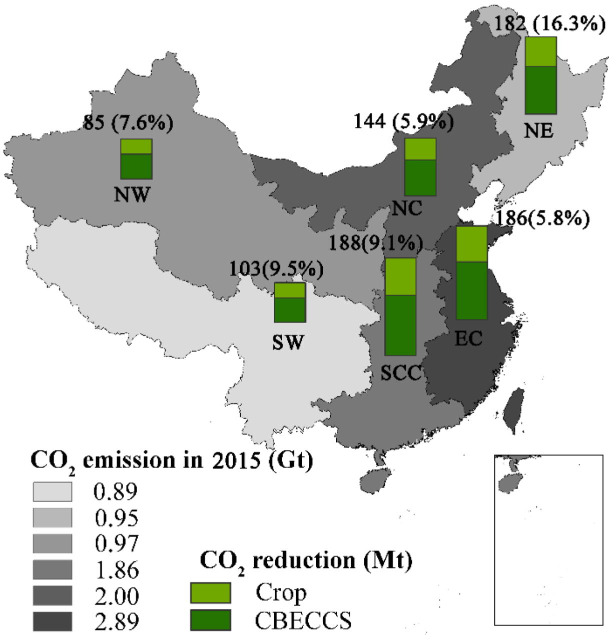

Fig. S5 Reductions in annual total CO_2 emission achieved by the by the CBECCS-CrB2 with a mixing ratio of biomass of 35%.

References

1. Metz B, Davidson O, Coninck Hd, Loos M, & Meyer L (2005) Carbon Dioxide Capture and Storage. *IPCC Special Report*.
2. Cormos C-C (2010) Evaluation of energy integration aspects for IGCC-based hydrogen and electricity co-production with carbon capture and storage. *International Journal of Hydrogen Energy* 35(14):7485-7497.
3. Chen Q, Rao A, & Samuelsen S (2014) H2 coproduction in IGCC with CCS via coal and biomass mixture using advanced technologies. *Applied Energy* 118:258-270.
4. Larson ED*, et al.* (2010) Co-production of decarbonized synfuels and electricity from coal + biomass with CO2capture and storage: an Illinois case study. *Energy Environ. Sci.* 3(1):28-42.
5. Jin HG, Wood A, & Seiler A (2015) Roadmap for Carbon Capture and Storage Demonstration and Deployment In The People's Repubic of China. *ASIAN DEVELOPMENT BANK* (https://www.adb.org/publications/roadmap-carbon-capture-and-storage-demonstration-and-deployment-prc).
6. Li X*, et al.* (2009) CO2 point emission and geological storage capacity in China. *Energy Procedia* 1(1):2793-2800.
7. Corti A & Lombardi L (2004) Biomass integrated gasification combined cycle with reduced CO2 emissions: Performance analysis and life cycle assessment (LCA). *Energy* 29(12-15):2109-2124.
8. Boerrigter H & van der Drift A (2004) Description of R&D trajectory necessary to reach large-scale implementation of renewable syngas from biomass. *ECN* 112:1-29.
9. Prins MJ, Ptasinski KJ, & Janssen FJJG (2006) More efficient biomass gasification via torrefaction. *Energy* 31(15):3458-3470.
10. Valero A & Usón S (2006) Oxy-co-gasification of coal and biomass in an integrated gasification combined cycle (IGCC) power plant. *Energy* 31(10-11):1643-1655.
11. Devi L, Ptasinski KJ, & Janssen FJJG (2003) A review of the primary measures for tar elimination in biomass gasi cation processes. *Biomass and Bioenergy* 24:125–140.
12. Asadullah M (2014) Barriers of commercial power generation using biomass gasification gas: A review. *Renewable and Sustainable Energy Reviews* 29:201-215.
13. Emun F, Gadalla M, Majozi T, & Boer D (2010) Integrated gasification combined cycle (IGCC) process simulation and optimization. *Computers & Chemical Engineering* 34(3):331-338.
14. Tim Fout*, et al.* (2015) Cost and Performance Baseline for Fossil Energy Plants Volume 1b_ Bituminous Coal (IGCC) to Electricity Revision 2b – Year Dollar Update. *National Energy Technology Laboratory (NETL).* DOE/NETL-2015/1727.
15. Fout T*, et al.* (2015) Cost and Performance Baseline for Fossil Energy Plants Volume 1b: Bituminous Coal (IGCC) to Electricity. *National Energy Technology Laboratory* https://www.netl.doe.gov/File%20Library/Research/Energy%20Analysis/Publications/Rev-2b-Vol-1b-IGCC_final.pdf.
16. Jacob C (2006) A scent of therapy: pharmacological implications of natural products containing redox-active sulfur atoms. *Nat Prod Rep* 23(6):851-863.
17. Eowa JS (2002) Recovery of Sulfwr from Sour Acid Gas: A Review of the Technology. *Environmental Progress* 21:143-162.
18. Giuffrida A, Romano MC, & Lozza GG (2010) Thermodynamic assessment of IGCC power plants with hot fuel gas desulfurization. *Applied Energy* 87(11):3374-3383.

19. Liao C, Yan Y, Wu C, & Huang H (2004) Study on the distribution and quantity of biomass residues resource in China. *Biomass and Bioenergy* 27(2):111-117.
20. Zhou X*, et al.* (2011) Assessment of sustainable biomass resource for energy use in China. *Biomass and Bioenergy* 35(1):1-11.
21. Gao J*, et al.* (2016) An integrated assessment of the potential of agricultural and forestry residues for energy production in China. *GCB Bioenergy* 8(5):880-893.
22. Liao C, Yan Y, Wu C, & Huang H (2004) Study on the distribution and quantity of biomass residues resource in China. *Biomass and Bioenergy* 27(2):111-117.
23. Lv P, Wu C, Ma L, & Yuan Z (2008) A study on the economic efficiency of hydrogen production from biomass residues in China. *Renewable Energy* 33(8):1874-1879.
24. Liao C, Wu C, Yan Y, & Huang H (2004) Chemical elemental characteristics of biomass fuels in China. *Biomass and Bioenergy* 27(2):119-130.
25. Jiang D, Zhuang D, Fu J, Huang Y, & Wen K (2012) Bioenergy potential from crop residues in China: Availability and distribution. *Renewable and Sustainable Energy Reviews* 16(3):1377-1382.
26. National Bureau of Statistics of China(NBSC) (2016) Output of major farm product. *Available at http://data.stats.gov.cn/english/easyquery.htm?cn=C01*.
27. Liu H, Jiang G, Zhuang H, & Wang K (2008) Distribution, utilization structure and potential of biomass resources in rural China: With special references of crop residues. *Renewable and Sustainable Energy Reviews* 12(5):1402-1418.
28. Yang Q, Han F, Chen Y, Yang H, & Chen H (2016) Greenhouse gas emissions of a biomass-based pyrolysis plant in China. *Renewable and Sustainable Energy Reviews* 53:1580-1590.
29. Yang Y*, et al.* (2010) Quantitative appraisal and potential analysis for primary biomass resources for energy utilization in China. *Renewable and Sustainable Energy Reviews* 14(9):3050-3058.
30. Piao S*, et al.* (2010) The impacts of climate change on water resources and agriculture in China. *Nature* 467:43.
31. Wang J, Wang E, Yang X, Zhang F, & Yin H (2012) Increased yield potential of wheat-maize cropping system in the North China Plain by climate change adaptation. *Climatic Change* 113(3):825-840.
32. Yue D, You F, & Snyder SW (2014) Biomass-to-bioenergy and biofuel supply chain optimization: Overview, key issues and challenges. *Computers & Chemical Engineering* 66:36-56.
33. Acharya B, Sule I, & Dutta A (2012) A review on advances of torrefaction technologies for biomass processing. *Biomass Conversion and Biorefinery* 2(4):349-369.
34. Yang J*, et al.* (2014) Potential usage, vertical value chain and challenge of biomass resource: Evidence from China’s crop residues. *Applied Energy* 114:717-723.
35. Zhang Q, Zhou D, Zhou P, & Ding H (2013) Cost Analysis of straw-based power generation in Jiangsu Province, China. *Applied Energy* 102:785-793.
36. Hao H, Geng Y, Li W, & Guo B (2015) Energy consumption and GHG emissions from China's freight transport sector: Scenarios through 2050. *Energy Policy* 85:94-101.
37. Zhao X., Tan Z., & Liu P. (2013) Development goal of 30GW for China’s biomass power generation: Will it be achieved? *Renewable and Sustainable Energy Reviews* 25:310-317.
38. Kreutz TG, Larson ED, Liu G, & Williams RH (2008) Fischer-Tropsch Fuels from Coal and

Biomass. *25th Annual International Pittsburgh Coal Conference*.
39. Liang XY*, et al.* (2013) Up-to-date life cycle assessment and comparison study of clean coal power generation technologies in China. *Journal of Cleaner Production* 39:24-31.
40. Xiong CG, Xie ZQ, & WY Y (2010) Study on Basic Characteristics of Crop Straw/Stalk Used as Biomass Energy Resources. *Southwest China Journal of Agricultural Sciences* 23:1725-1732.
41. Liang Q*, et al.* (2012) Effects of 15 years of manure and inorganic fertilizers on soil organic carbon fractions in a wheat-maize system in the North China Plain. *Nutrient Cycling in Agroecosystems* 92(1):21-33.
42. Tang H, Qiu J, Van Ranst E, & Li C (2006) Estimations of soil organic carbon storage in cropland of China based on DNDC model. *Geoderma* 134(1):200-206.
43. Kahrl F*, et al.* (2010) Greenhouse gas emissions from nitrogen fertilizer use in China. *Environmental Science & Policy* 13(8):688-694.
44. Metz B, Davidson OR, Bosch PR, Dave R, & Meyer LA (2007) Climate Change 2007: Mitigation. Contribution of Working Group III to the Fourth Assessment Report of the Intergovernmental Panel on Climate Change. *Cambridge University Press, New York, NY*.
45. Garnett T (2011) Where are the best opportunities for reducing greenhouse gas emissions in the food system (including the food chain)? *Food Policy* 36:S23-S32.
46. Xu HL (2012) Investigation on fertilization status and analysis on effect of formula fertilization for rape in Yangtze River Basin *Huazhong Agricultural University*.
47. Luo WB, Deng MJ, & GC X (2015) Study on the Space-Time Evolution and Reduction Potential of Carbon Emission in Chinese Cotton Planting Fertilization *Economic Geography* 35:149-156.
48. Ye YL, Han YL, & Tan JF (2007) Wheat Production and Fertilizer Application in China *Journal of Triticeae crops* 27:127-133.
49. Jiang XY, Fan ZQ, & S G (2014) Study on Caloric Value of Crop Seeds and Various Crop Organs in Shandong Province *Chinese Agricultural Science Bulletin* 30:109-113.
50. Kumar A & Sokhansanj S (2007) Switchgrass (Panicum vigratum, L.) delivery to a biorefinery using integrated biomass supply analysis and logistics (IBSAL) model. *Bioresource Technology* 98(5):1033-1044.
51. Emery IR & Mosier NS (2012) The impact of dry matter loss during herbaceous biomass storage on net greenhouse gas emissions from biofuels production. *Biomass and Bioenergy* 39:237-246.
52. Weisser D (2007) A guide to life-cycle greenhouse gas (GHG) emissions from electric supply technologies. *Energy* 32(9):1543-1559.
53. Zhang R (2006) Mining Engineering Design Handbook. *Coal Industry Press*.
54. Huang S & Dou Q (2007) China coal industry yearbook 2006. *China Coal Information Institute, Coal Industry Press*.
55. CTYH (2007) China's Traffic Yearbook 2006. *China's Traffic Yearbook House, Beijing*.
56. Xia D, Ren Y, & Shi L (2010) Measurement of Life-Cycle Carbon Equivalent Emissions of Coal-Energy Chain. *Statistical Research* 27(8):82-89.
57. CEPEI (2011) Thermal Power Engineering Design Handbook 2010. *China Power Engineering Consulting Group Corporation, China Electric Power Press, Beijing*.
58. Timothy S & Robert J (2010) Life Cycle Analysis: Integrated Gasification Combined Cycle (IGCC) Power Plant. *National Energy Technology Laboratory*
59. Catalanotti E, Hughes KJ, Porter RTJ, Price J, & Pourkashanian M (2014) Evaluation of

performance and cost of combustion-based power plants with CO2 capture in the United Kingdom. *Environmental Progress & Sustainable Energy* 33(4):1425-1431.

60. Basu P, Butler J, & Leon MA (2011) Biomass co-firing options on the emission reduction and electricity generation costs in coal-fired power plants. *Renewable Energy* 36(1):282-288.

61. Hedenus F, Karlsson S, Azar C, & Sprei F (2010) Cost-effective energy carriers for transport – The role of the energy supply system in a carbon-constrained world. *International Journal of Hydrogen Energy* 35(10):4638-4651.

62. Matuszewski M (2012) Greenhouse Gas Reductions in the Power Industry Using Domestic Coal and Biomass Volume 1: IGCC. *National Energy Technology Laboratory* Source: https://www.netl.doe.gov/research/energy-analysis/search-publications/vuedetails?id=754.

63. Qinhuangdao coal network (2018) Bohai rim power coal price. *http://www.cqcoal.com/exp/exponent.jsp*.

64. Siefert NS & Litster S (2013) Exergy and economic analyses of advanced IGCC–CCS and IGFC–CCS power plants. *Applied Energy* 107:315-328.

65. Zhuang J, Liang Z, Lin T, & Guzman FD (2007) Theory and Practice in the Choice of Social Discount Rate for Cost-Benefit Analysis: A Survey *Asian Development Bank*.

66. Ni H*, et al.* (2015) Emission characteristics of carbonaceous particles and trace gases from open burning of crop residues in China. *Atmospheric Environment* 123:399-406.

67. Sillapapiromsuk S, Chantara S, Tengjaroenkul U, Prasitwattanaseree S, & Prapamontol T (2013) Determination of PM10 and its ion composition emitted from biomass burning in the chamber for estimation of open burning emissions. *Chemosphere* 93(9):1912-1919.

68. Huang X, Li M, Li J, & Song Y (2012) A high-resolution emission inventory of crop burning in fields in China based on MODIS Thermal Anomalies/Fire products. *Atmospheric Environment* 50:9-15.

69. Fu X*, et al.* (2013) Emission inventory of primary pollutants and chemical speciation in 2010 for the Yangtze River Delta region, China. *Atmospheric Environment* 70:39-50.

70. Akagi SK*, et al.* (2011) Emission factors for open and domestic biomass burning for use in atmospheric models. *Atmospheric Chemistry and Physics* 11(9):4039-4072.

71. Rohde RA & Muller RA (2015) Air Pollution in China: Mapping of Concentrations and Sources. *PLoS One* 10(8):e0135749.

72. Lelieveld J, Evans JS, Fnais M, Giannadaki D, & Pozzer A (2015) The contribution of outdoor air pollution sources to premature mortality on a global scale. *Nature* 525(7569):367-371.

73. Ji D*, et al.* (2014) The heaviest particulate air-pollution episodes occurred in northern China in January, 2013: Insights gained from observation. *Atmospheric Environment* 92:546-556.

74. Sultana A & Kumar A (2011) Optimal configuration and combination of multiple lignocellulosic biomass feedstocks delivery to a biorefinery. *Bioresource Technology* 102(21):9947-9956.

75. Wang C, Zhang L, Chang Y, & Pang M (2015) Biomass direct-fired power generation system in China: An integrated energy, GHG emissions, and economic evaluation for Salix. *Energy Policy* 84:155-165.

76. Peng HJ*, et al.* (2006) Feasibility report of Jianhu biomass gasification project. Feasibility report of Jianhu biomass gasification project. *Nanjing Guolian Electric Power Engineering Design Co. LTD*.

77. Ancora MP, Zhang L, Wang S, Schreifels J, & Hao J (2015) Economic analysis of atmospheric

mercury emission control for coal-fired power plants in China. *Journal of Environmental Sciences* 33:125-134.

78. Cai S*, et al.* (2017) The impact of the "Air Pollution Prevention and Control Action Plan" on PM2.5 concentrations in Jing-Jin-Ji region during 2012–2020. *Science of The Total Environment* 580:197-209.
